# Design Principles for Interactive Dashboards in Drug Safety Surveillance: Design Science Research

**DOI:** 10.2196/75936

**Published:** 2026-02-27

**Authors:** Malwina Kotowicz, Mexhid Ferati, Soumitra Chowdhury, Cláudio Martins Pires

**Affiliations:** 1 Department of Informatics, Faculty of Technology, Linnaeus University, Växjö, 351 95, Sweden, 46 470767999

**Keywords:** drug safety surveillance, adverse drug reactions, pharmacovigilance, dashboard systems, medical informatics applications, design science research, decision support systems, user-centered design, clinical

## Abstract

**Background:**

Adverse drug reactions pose a serious threat to health care, leading to patient harm and substantial economic burden. Dashboards for drug safety surveillance are a valuable tool to tackle it.

**Objective:**

This qualitative study aims to develop a dashboard for drug safety tracking with active and iterative involvement of end users. To support dashboard development, we formulate and iteratively refine design principles (DPs) for drug safety dashboards using the affordance theory.

**Methods:**

Following a design science research approach, we conducted 3 cycles of iterative design and evaluation involving the end users. Four professional end users (with expertise in drug screening, drug discovery, and data science) and 6 nonprofessional end users (drug consumers) were engaged in the requirements gathering sessions through co-design workshops, usability testing through think-aloud sessions, and heuristic evaluation.

**Results:**

The analysis resulted in a set of 8 DPs refined using the prototype’s affordances (ie, actionable properties of the dashboard that guide user interaction and interpretation of data). Following themes emerged in the formulation and refinement of DPs: addressing the bootstrap problem through designing for immediate use (DP1a), allowing identification of patterns through visualizing causality while signalizing uncertainty (DP1b), tracking trends for relevant variables (DP1c), implementing user-controlled views (DP2a) and customizable levels of data granularity (DP2b), guiding user’s visual attention through spatial layouts (DP2c), designing for higher public value (DP3a) and providing features to support decision-making for varied stakeholders’ groups (DP3b). A high-fidelity dashboard prototype for drug safety surveillance was proposed as a result of applying the final set of DPs. Heuristic evaluation of the prototype revealed an overall usability score of 84%.

**Conclusions:**

Applying DPs rooted in affordance theory led to a purposeful and user-relevant artifact that can improve understanding of drug safety data and potentially guide decision-making processes for professional users. Our theoretical contribution lies in providing refined DPs, while also demonstrating how affordances can aid dashboard development in pharmacovigilance. Our findings may be applicable to similar health information systems in related domains.

## Introduction

### Overview

Adverse drug reactions (ADRs) pose a serious risk, leading to hospitalization of millions of patients every year, and the incidence of ADRs resulting in hospitalization has been consistently increasing in recent years [1-5]. ADRs are not only a major health concern, but also a tremendous economic burden, generating costs of several billion dollars each year [6,7]. Monitoring side effects associated with medicinal products is critical for regulatory bodies, health care providers, pharmacists, and, most importantly, drug consumers.

The rapid spread of health misinformation through online channels poses significant risks to public health [8,9]. Evidence suggests that many individuals, including physicians and medical students, resort to online searches for clinical information (ie, general web and social media queries rather than official pharmacovigilance portals). These channels frequently present user-generated or commercially sponsored content (eg, and influencer posts) that lacks expert review. Only a fraction of users consult credible resources (eg, specialized academic databases) [10,11]. Importantly, more than half of the online searches by health care professionals focus on drug safety and ADRs [12]. Relying on unverified sources for health information can be a major threat to public health, erode public trust, and can lead to severe consequences, including harm or death [13]. Specialized platforms, such as interactive visualization dashboards for drug safety information at-a-glance, offer drug safety information in a trusted and concise way. Such platforms could be intended for professional users (ie, health care professionals, pharmacists, researchers, pharmaceutical companies, and regulatory agencies concerned with pharmacovigilance), as well as nonprofessional users (ie, patients, medical journalists, or lawyers protecting patient rights).

Dashboards in pharmacovigilance (DiPs), however, are scarce and often have complex interfaces resembling decision support systems rather than accessible designs. Additionally, most of the DiPs are not available online as open-source platforms, but are offered as proprietary software under license [14-17]. As a result, they have a limited audience and lack widespread availability. Moreover, DiP often lacks salient usability features, is poorly designed due to the absence of a structured design approach, or has functional deficiencies [17,18]. Several data mining techniques have been proposed as a valuable tool for early detection of potential safety signals (ie, indications or evidence that suggest a possible new ADR) in pharmacovigilance [19,20]. However, existing DiP rarely integrates data mining methods to enhance raw drug safety information and therefore offers limited insight [21-23]. Given the shortcomings of current platforms for drug safety surveillance and the scarcity of prescriptive knowledge regarding their design, we investigate the idea of finding more fitting designs for handling such data. In search of suitable design principles (DPs) that should lead the development of similar platforms, this study relies on the design science research (DSR) strategy and is guided by the following research question: what are the appropriate DPs for interactive dashboards in drug safety surveillance?

To address this question, we set out to develop and evaluate DPs for a class of dashboards in pharmacovigilance, following the DSR approach. We show that engaging in prototype creation reveals general DPs applicable to similar systems. Consequently, besides the artifact being developed, emergent DPs are proposed as transferable concepts for similar systems within the boundary conditions studied. To this end, we use the notion of affordances—properties of physical things or relationships between actors and these things—to support our research. Affordances help in exploring different designs in this study, since different physical features of the prototype can afford (ie, provide and bring about) the same functionality.

This study consists of the following steps. First, we identify general DPs for visual dashboards of drug safety data. Second, following the DSR methodology, we perform the requirements gathering session with a group of experts in drug screening, bioinformatics, and data science. Third, we create a prototype of an interactive dashboard, where we present drug safety data in a visually friendly manner, with the purpose of improving its general understandability. To this end, we also focus on finding suitable data mining techniques for knowledge extraction from publicly available data in pharmacovigilance. Fourth, we assess the prototype in demonstration and evaluation sessions with users to detect potential usability issues and receive feedback on possible improvements to the design. Altogether, we carry out 3 iterations of the DSR phases.

The aim of this research is thus to enhance the understanding of drug safety data and improve the potential for decision-making among intended users. Specifically, our objectives are to (1) formulate DPs, (2) re-evaluate these in 3 iterations of alternating phases of prototype development, demonstration, and evaluation, and (3) gain insight on how the notion of affordances and DPs can support the development of visualization tools in drug safety surveillance. It is important to mention that, in this study, by “professional users” we refer specifically to researchers in drug screening, bioinformatics, and data science; clinicians and pharmacists were not recruited in our sample. Accordingly, the DPs and evaluation results reported here are grounded in researcher workflows. While some principles are likely cross-cutting across stakeholder groups, we do not evaluate their applicability to clinician- or pharmacist-facing workflows in this study.

### Current State of Drug Safety Surveillance Systems and Data Mining Standards

Three major global databases monitor drug safety: Food and Drug Administration (FDA) Adverse Events Reporting System (FAERS) database, VigiBase maintained by the Uppsala Monitoring Center (UMC) and World Health Organization (WHO), and EudraVigilance managed by the European Medicines Agency (EMA). FAERS, the world’s largest repository with more than 24 million reports as of March 2022, serves as a key postmarketing pharmacovigilance tool. EudraVigilance and VigiBase similarly collect ADR reports from the European Economic Area. These reports, submitted by manufacturers (mandatory) and health care professionals or consumers (voluntary), document suspected side effects from one or more drugs per patient case [20,24,25].

Although crucial for surveillance, these reports cannot establish direct causality because of factors such as comorbidities or concurrent medication [25]. To address this, database curators apply data mining to detect safety signals for expert review. The FDA encourages signal detection in drug development, as it positively impacts the process, while the WHO and UMC offer similar services to industry partners [20,26].

Several digital platforms exist for drug safety surveillance, but many, including FAERS, rely solely on reporting frequencies rather than integrated data mining [21]. According to Kumar [17], the FAERS dashboard is affected by limited awareness, unclear purpose, unintuitive navigation, and challenges in interpreting its data. Most reviewed platforms pose accessibility challenges, as they rely on proprietary databases, restricting access, reproducibility, and public transparency [16]. Many are outdated or limited in scope, relying on small datasets or alternative data sources instead of major repositories [14-16,27,28], while others focus only on ADR reporting rather than analysis and visualization [29-31].

Moreover, most platforms were not developed using the DSR process or participatory design methods and lack usability testing. Therefore, their complex interfaces often resemble expert decision support systems rather than accessible designs [14-16], which can impact usability, hinder tool adoption, and compromise their practical impact [18,32]. These factors emphasize the need for open-source, user-centered drug safety tools that enhance accessibility and stakeholder engagement.

Data mining techniques are routinely used in pharmacovigilance to enhance the understanding of drug and vaccine safety profiles [33-36]. While no universal standard exists, guidelines provide statistical support for interpreting detected signals [37]. Disproportionality analysis (DPA) is currently the most common technique recommended by the FDA, UMC (WHO), and EMA, although the organizations deploy different statistical approaches [38]. Despite its importance, DPA results are seldom integrated into public dashboards, which continue to rely mainly on reporting frequencies [21-23]. This limitation may hinder a thorough understanding of the drug safety profiles and potentially lead to overlooking important trends, which further emphasizes the importance of incorporating safety signal assessments in public dashboards in pharmacovigilance.

New platforms have been developed in recent years primarily leveraging artificial intelligence (AI), machine learning, and real-world data integration for ADR detection in clinical practice. Specific new platforms include the PVClinical system using OMOP-CDM and Observational Health Data Sciences and Informatics (OHDSI) software stack for electronic health record integration [39] and established platforms such as Oracle Argus Safety, repClinical, PvNET, and ArisG for ADR administration and reporting [40]. AI-powered approaches show particular promise, with systematic reviews demonstrating effective natural language processing application across 16 studies for user-generated content analysis [41] and 12 studies showing deep learning models outperforming traditional machine learning for adverse event extraction [42]. However, challenges remain, including data quality, regulatory compliance, and the need for clinical validation of these emerging platforms [43].

Recent research on human-computer interaction (HCI) and user-centered design in pharmacovigilance dashboards is limited but emerging, with only a few directly relevant studies identified in the past few years. The strongest evidence comes from Yu et al [44] who developed a visualization platform for FDA adverse event data with usability demonstration through case studies and validated accuracy against manually processed data. In a study on integrating human-centered design of a public health data dashboard for sexually transmitted infections in the state of New York, Ansari and Martin [45] found that dashboards must reflect actual workflow needs, address data limitations transparently, and use design elements, such as clear notes and estimated data displays, to build user trust and handle common data quality issues. This process resulted in a highly functional design template. Malkani et al [46] identified and ranked key health care dashboard design attributes such as clarity, simplicity, visual hierarchy, responsiveness, and intuitive navigation as most critical for improving usability and user-centered decision-making. Gavriilidis et al [47] applied user-centered DPs to create the PVClinical platform for ADR investigation, identifying “actionability” as a key adoption factor and emphasizing needs for explainable, human-interpretable results. Broader context comes from patient safety dashboard research, where Murphy et al [48] found that among 33 studies, only 4 incorporated informatics or human factors principles in development or evaluation, highlighting a significant gap in rigorous HCI application.

The evidence base remains thin, with most pharmacovigilance visualization research focusing on technical implementation rather than systematic user-centered design evaluation. More rigorous HCI research is needed in this specialized domain [44,47,48]. Table 1 summarizes the limitations of available dashboards and shows the prototype features we explore in response to the identified gaps.

**Table 1.** Research gaps were identified through limitations to available dashboards for pharmacovigilance and prototype features were intended to address them.

Platform category (examples)	Key limitations noted in the literature	Intended prototype features
Regulatory public dashboards (FAERS^a^; EudraVigilance; VigiLyze)	Emphasize reporting frequencies; statistical signal detection (eg, DPA)^b^ typically not available in the public UI^c^; access and reproducibility constraints (open access but not open source; VigiLyze requires membership). The FAERS public dashboard shows issues with awareness, navigation, and interpretability for nonspecialists [17,21-23]	Integrates DPA outputs in the UI; offers guided interactions; co‑designed and usability‑tested; open data and code with an open‑access dashboard
Enterprise pharmacovigilance dashboards (Oracle Argus Safety; ArisG; repClinical; PvNET)	Proprietary, case‑management and regulatory‑reporting focus; limited public documentation of integrated analytics, no participatory design or formal usability evaluation; constrained transparency and reproducibility [40]	Offers exploratory analysis; transparent metrics (downloadable DPA files); hybrid‑open model enabling inspection, reuse, and migration to a fully open‑source stack
Clinical and EHR analytics on OMOP‑CDM (PVClinical)	Integrates EHRs^d^ via the OHDSI^e^ stack and reports user‑centered design; scope differs from spontaneous report dashboards; platform availability varies; continued need for clinical validation and regulatory alignment [39,43,47]	Complements PVClinical by focusing on FAERS spontaneous reports; reports outcomes of co‑design, think‑aloud usability (and its quantitative outcomes), and heuristic evaluation as a result of systematic user involvement in multicycle evaluation process
Academic or research visualization platforms	Often older or limited‑scope implementations with small or alternative datasets; few report systematic user-centered design [14-16,28,44]	Built on open FAERS data—the largest, regularly updated public repository with API^f^ access; applies recent visualization techniques via implementation of domain‑informed design principles
Patient‑facing ADR^g^ reporting and medication information apps	Designed primarily for ADR submission or mobile information; not intended for exploratory signal analytics or integrated DPA visualization [27,29-31]	Analytic dashboard for exploring suspected drug-event patterns, supporting stakeholder decision contexts

a FAERS: Food and Drug Administration Adverse Events Reporting System.

b DPA: disproportionality analysis.

c UI: user interface.

d EHR: electronic health record.

e OHDSI: Observational Health Data Sciences and Informatics.

f API: application programming interface.

g ADR: adverse drug reaction.

### Affordance as a Lens to Study DPs

Affordances are the action possibilities an artifact enables through its material features [49-51]. We use this lens operationally: each DP states (1) the intended user activity (affordance), (2) the dashboard features that enable it (material properties), and (3) the boundary conditions in which the principle applies. In this study, the artifact does not itself cause understanding or decisions; it supports them insofar as its features enable the specified affordances [52].

### Formulating the DPs

DPs provide prescriptive guidance for constructing an artifact to meet stated objectives [50,53-55]. Drawing on literature on pharmacovigilance data literacy and dashboard design, we formulated DPs using the structure by Chandra et al [53] that ties (1) intended user activity (affordance), (2) enabling features (material properties), and (3) boundary conditions. We use the following template for DPs formulation:

Provide the system with (material property—in terms of form and function) in order for users to (activity of user or group of users—in terms of action) given that (boundary conditions—user group’s characteristics or implementation settings) [53].

We consider drug safety surveillance as the boundary condition and analyze 2 user groups for this DSR prototype: (1) nonprofessionals (drug consumers) and (2) professional users as operationalized in this study (researchers in drug screening, bioinformatics, and data science). While health care professionals and pharmacists are central stakeholders for pharmacovigilance dashboards, they were not recruited in the present sample; consequently, findings and DPs are grounded in these boundary conditions.

We categorize the DPs into three classes: (1) DPs related to functionality that address the question of how well the dashboard serves its purpose, (2) DPs related to the mode of display that refer to the specific visualization techniques, and (3) DPs related to added value, which focus on the benefits the artifact provides to the public or end users.

We selected and refined DPs by applying an affordance alignment test: a principle was retained only when a target user goal could be mapped to a concrete dashboard feature expected to enable the activity in the drug safety context [52,53]. Subsequent evaluation rounds confirmed and sharpened their scope.

#### DPs Related to Functionality

The value of new information infrastructures (IIs) lies in the users’ community. However, since this community is initially small, the benefits of using the system may not be immediately apparent. Several authors emphasized the bootstrap problem that arises when the benefits of new IIs are perceived as indirect, delayed, or uncertain, which can lead to resistance in the system’s adoption [56,57]. To address this issue, Hanseth and Lyytinen [57] recommend designing IIs for direct usefulness, that is, prioritizing immediate practical benefits for the users. This approach may enhance the perceived value of the system and help expand the user community. It may also create a positive feedback loop, where the use of the system leads to its further improvements, which in turn increase its usefulness and value. Consequently, we formulated the following DP: DP1a: it provides features to address the bootstrap problem, so that the system affords immediate use of the dashboard in drug safety surveillance.

Recker [58] investigates public dashboards related to the coronavirus pandemic, concluding that many present data through simple attributes (eg, number of cases, number of deaths, and number of recovered). Integrating state-tracking features that enable monitoring changes in states and events that trigger them (such as fluctuations in infection rates, or timelines of lockdowns and restrictions) could be a significant improvement, and the information presented in dashboards should answer the question how, instead of just how many. We formulated two DPs: (1) DP1b: it provides features to describe possible causality between variables, so that the system affords identification of patterns and trends in drug safety surveillance. (2) DP1c: it provides features to track changes in variables, so that the system affords tracking of the progression of relevant states in drug safety surveillance.

#### DPs Related to the Mode of Display

Matheus et al [59] suggest incorporating customizable views into decision-making dashboards, as this design strategy may help bypass the interpretation bias—“a single view might result in a limited picture of the situation.” Expanding views to more than one view may lead to better understanding of the problem. Customized views help users to focus on relevant information and reduce the cognitive load associated with processing large volumes of data. We formulated the following DP: DP2a: it provides features to support customized views, so that the system affords examining various dimensions of data in drug safety surveillance.

Matheus et al [59] also suggest incorporating customizable features into decision-making dashboards, including the ability to toggle between detailed and high-level data visualizations. As these dashboards often contain a large volume of data, such features can help users understand the problem at hand by providing varying levels of detail. We formulated the following DP: DP2b: it provides overview and details, so that the system affords exploring data of different granularity levels in drug safety surveillance.

Toreini et al [60] follow a set of experiments to find ways to decrease task resumption failures. Tracking the real-time eye movements of the participants, they conclude that users start from and focus their attention on the upper left corner of the dashboard. Several authors suggest incorporating attention-augmenting features into dashboard elements (such as varied color, size, shape, and orientation) and visual cues (buttons and dials) to direct attention toward objects of higher importance [60,61] Consequently, DP2c: it provides features that stimulate attention and place important elements on the top-left area of the dashboard, so that the system affords improved attention management while using the dashboard in drug safety surveillance.

#### DPs Related to Added Value

Matheus et al [59] claim that creating dashboards should go beyond simply visualizing data. Creating public value should be embedded in the design process, as the data on its own is of little use. This way of thinking of the design process—through the prism of public value—transgresses the initial idea of merely finding the ways to present data in the best visual way. Data visualization should be the means, not the goal, and asking the question “what is the added value?” during the design process is necessary. Designers should prioritize generating public values such as engagement, transparency, and accountability, while also upholding public values such as privacy. Consequently: DP3a: it provides features to create public values, so that the system affords users’ engagement in dashboard use in drug safety surveillance.

Embedding features that support decision-making is a crucial aspect of dashboard development [59]. Such features can be valuable for professional users, including health care professionals, pharmacists, researchers, and regulatory bodies in pharmacovigilance. We also expect the users would gain greater understanding and take up higher responsibility toward their own health, which would equip them better for decision-making. We formulated the following DP: DP3b: it provides features to support decision-making, so that the system affords users to evaluate what-if scenarios and consider different alternatives in drug safety surveillance. Initial DPs are shown in Table 2.

**Table 2.** Overview of initial design principles.

Design principle	Affordance	Material properties
DP1a	Obtaining immediate practical benefits while using the dashboard [56]	Features that respond to users’ requirements, features that address actual user problems and offer solutions
DP1b	Identification of patterns and trends through exploration of DPA^a^ analysis [58]	Features that enable identification and examination of patterns and trends in drug-ADR^b^ evaluations
DP1c	Monitoring the progression of relevant states (how variables change over time) [58]	Features that allow tracking changes in relevant variables
DP2a	Exploring, analyzing, and interpreting data from different angles [59]	Features that enable analysis of data from multiple perspectives
DP2b	Exploring data from a broad and narrow perspective by switching between levels of granularity [59]	Features to adjust the level of detail depending on users’ needs and the context of analysis
DP2c	Improved attention management while using the dashboard [60,61]	Features that stimulate attention, visual cues that direct attention toward important elements
DP3a	Active involvement in exploring the data, potentially increasing users’ satisfaction [59]	Features with a potential to create public value (engagement, transparency, accountability, and adherence to privacy)
DP3b	Evaluating alternatives of actions, exploring what-if scenarios [59]	Features that support decision-making

a DPA: disproportionality analysis.

b ADR: adverse drug reaction.

## Methods

### Data Collection and Target Groups

**Figure 1.**
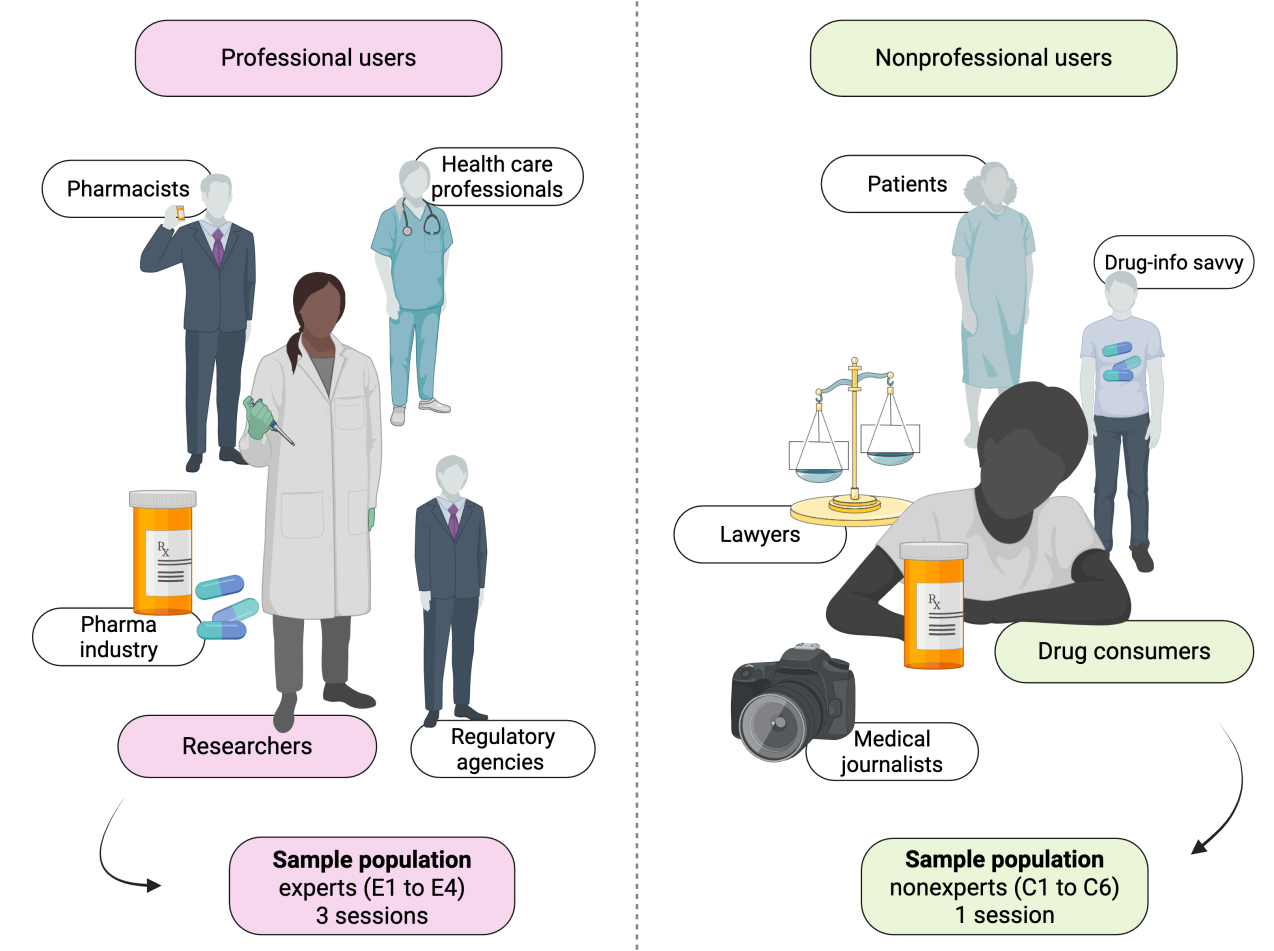
Professional (left) and nonprofessional (right) user groups. The broader professional stakeholder landscape includes health care professionals, pharmacists, researchers, regulatory agencies, and industry. In this study, the professional participants recruited were researchers in drug screening or drug development (E1-E4). Nonprofessional participants were drug consumers (C1-C6).

We have considered 2 target groups for this work: professional users and nonprofessional users. We define professional users as those whose professional activities are concerned with drug safety surveillance and thus have extensive knowledge in the domain. For clarity, throughout the paper, “professional users” refers to this researcher group; the implications for clinician- and pharmacist-facing workflows lie outside this study’s boundary conditions. In contrast, nonprofessional users are not professionally concerned by drug safety surveillance and in that sense are laymen in the domain of pharmacovigilance. Figure 1 depicts the user groups.

We have recruited a group of 4 expert users (referred to as experts, with participant IDs of E1 to E4) for this study. All experts were scientists of different academic degrees and expertise levels and were working on drug development and screening projects. Three out of 4 experts had extensive experience in data science and bioinformatics. In addition, all experts had many years of experience in data visualization and communication to the general and scientific community. Experts participated in the co-design workshop, pilot evaluation, and heuristic evaluation sessions.

For usability testing (think-aloud session), we have recruited a group of 6 nonexpert users (referred to as nonexperts, with participant ID of C1 to C6). All nonexperts were drug consumers and had a history of continuous medication therapy of at least 7 days at least once in the previous year. We assumed these users were potentially also drug safety information-savvy and might provide meaningful insights into which complex analyses are possible. Importantly, nonexperts were novices in the sense that they had no prior exposure to the tool in question or any other similar dashboards [62]. Although some nonexperts shared parts of professional experience with experts (eg, C4 and C5 had expertise in biomedical engineering and data science), they were not working on drug screening and discovery projects.

By design, think-aloud usability testing was conducted solely with nonprofessional “novice” users to evaluate first-time learnability and immediate practical use (DP1a) without prior-exposure effects from experts who had already interacted with the prototype during co-design and/or the pilot evaluation [62].

Overall, all participants were between 28 and 44 years old and were based in Germany, Sweden, France, Finland, and Portugal. A detailed description of participants recruited for this study is shown in Table S1 in Multimedia Appendix 1. Given the age range and the presence of multiple participants with advanced technical or professional backgrounds, this sample likely exhibits above-average digital and data literacy; while valuable for investigating complex interaction issues in early iterations, this may inflate task performance relative to older adults and users with lower digital health literacy.

### DSR Approach

We followed a framework proposed by Sauro and Lewis [63] to quantitatively analyze the user experience. Furthermore, in heuristic evaluation, we quantitatively collected and analyzed data in line with the framework proposed by Dowding and Merrill [64]. Additionally, we qualitatively collected and analyzed data in all experimental sessions in this study.

We modified a staged DSR model, initially proposed by Peffers et al [65]. Thus, the DSR framework applied in this study consisted of the following stages: (1) problem definition and objectives of the solution, (2) diagnosis phase, (3) data preparation phase, (4) design and development phase, and (5) demonstration and evaluation phase. Detailed descriptions of each phase are found in Multimedia Appendix 2.

**Figure 2.**
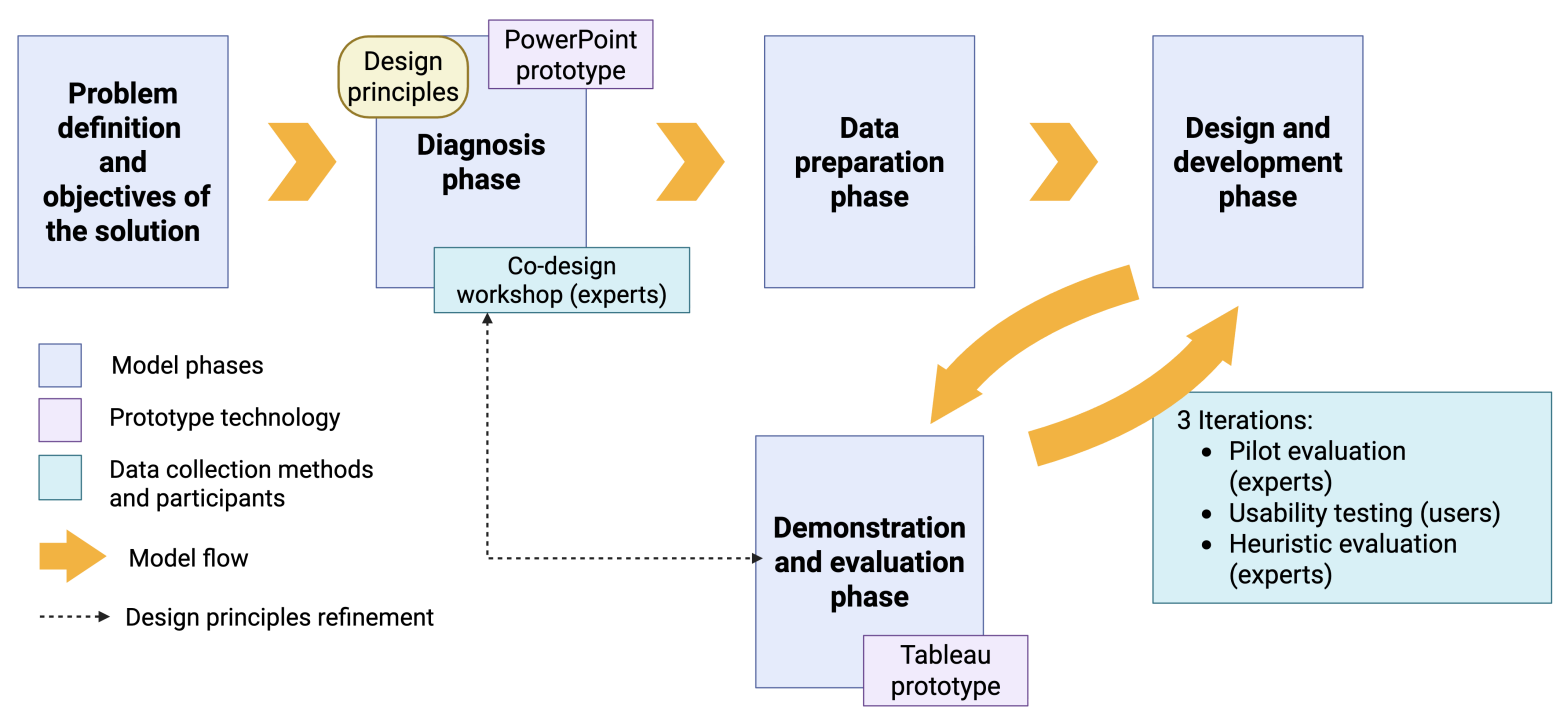
Applied design science research model.

A depiction of the DSR process is shown in Figure 2. v1-PowerPoint and v2-v4-Tableau prototypes were used in diagnosis and demonstration and evaluation phases, respectively. Blue boxes show individual phases and the order in which they occurred in DSR (yellow arrows). DPs emerged in the diagnosis phase and were then iteratively refined in the demonstration and evaluation phases.

### Data Collection in DSR Cycle

We have performed 3 iterations of the DSR cycle. That is, we started with the diagnosis phase, in which we first identified initial DPs by gathering secondary data available in the literature. Then, we proceeded with one requirement gathering session, in the form of a co-design workshop with experts. Next, we have engaged in the data preparation phase, where—after selecting a suitable drug safety data source—we have written Python scripts to get and process the data.

**Figure 3.**
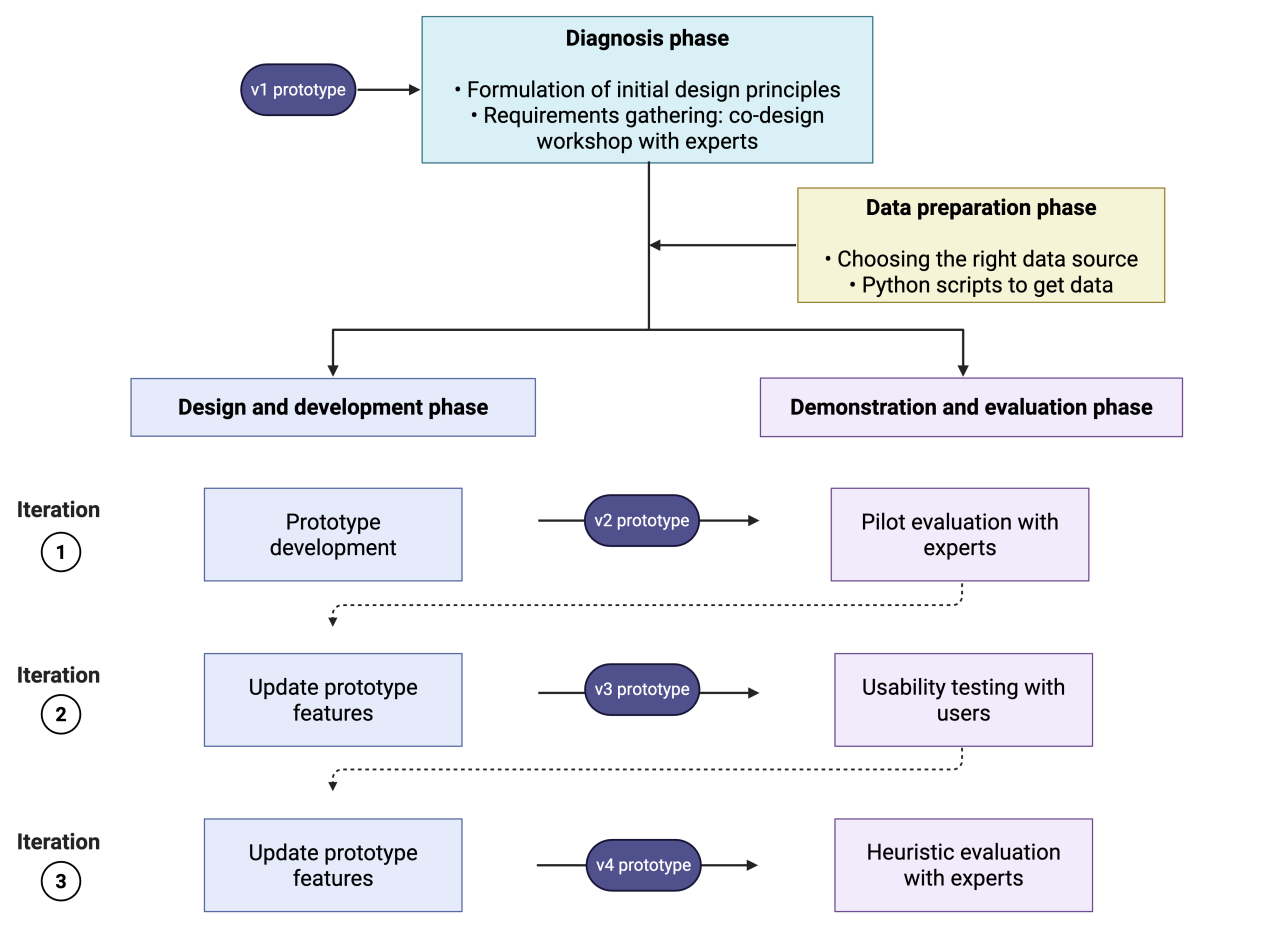
Overview of data collection activities.

We then performed 3 iterations of alternating design and development and demonstration and evaluation phases. In the first iteration, we developed a prototype in Tableau software (v2; Tableau Software LLC) [66], and tested it in the pilot evaluation session with experts. The results obtained in this way fed the second DSR iteration. We have updated prototype features (v3) and proceeded with usability testing (in a think-aloud session) with nonexperts. Finally, in the last iteration, upon updating prototype features (v4), we performed heuristic evaluation with experts. A flowchart of research activities is shown in Figure 3.

Further details about the design and development phase are provided in Multimedia Appendix 2. In that appendix, we describe the diagnosis phase, including the development of DPs and requirements gathering. We also provide information on how we used Statista [67] and participant requests to inform the selection process of 5 drugs used for prototype development: ibuprofen, acetaminophen (paracetamol), prednisone, quetiapine, and morphine.

### Demonstration and Evaluation Phase

#### Iteration 1: Pilot Evaluation

The researcher was moving through the dashboard by performing a set of predefined activities, while the participants controlled the navigation process by suggesting improvements whenever necessary. The session lasted 1 hour and was recorded with Otter.ai (Otter.ai, Inc) [68].

#### Iteration 2: Usability Testing

We have performed 6 usability tests with nonexpert users in think-aloud sessions. These included live meetings with 4 participants (C1, C2, C3, and C4) and virtual meetings with 2 participants (C5 and C6). Before the session, we have provided the users with a detailed protocol including introduction of the study, explanation on usability testing in the think-aloud session and a list with predefined tasks. These tasks required participants to do activities on the interface without the researchers’ guidance. For the most part, researchers remained silent during users’ task performance. Users were encouraged to express their thoughts while conducting the tasks. Additionally, 2 participants (C2 and C3) shared their thoughts on the usefulness of the dashboard and their interest in the tool in general. These short and unstructured discussions unfolded at the end of the sessions and were incorporated into findings. Each session lasted between 1.5 hours and 2.5 hours and was recorded with the same software, Otter.ai [68].

Experts were intentionally not included at this think-aloud stage to avoid learning effects from earlier sessions; their structured feedback was captured in the expert-focused co-design phase, pilot evaluation, and in the subsequent heuristic evaluation with 4 and 3 experts, respectively, which is within the 3‐5 evaluator range known to uncover the majority of usability problems [69,70].

#### Iteration 3: Heuristic Evaluation

As originally suggested by Nielsen [71] and confirmed by other authors [62,69,72,73], 85% of usability violations can be detected by 3 to 5 evaluators in heuristic evaluation. We have performed heuristic evaluation with 3 experts (E1, E2, and E3).

### Data Analysis

#### Overview

We used the framework method by Gale et al [74] to qualitatively analyze data in search of codes and themes. Importantly, and due to the specificity of DSR as a methodology (ie, when data are collected in iterations to repeatedly improve an artifact), codes and themes in this work refer to identified usability issues, dashboard features or elements undergoing evaluation and refinement, or any concept related to these (ie, the motivation behind the revision of DPs). Additionally, to prioritize the issues detected in usability testing and select appropriate solutions, we applied a method by Sauro and Lewis [63]. Finally, we analyzed the data from the heuristic evaluation through the assessment of 10 usability factors. These concepts are discussed further below. Overall, we detected 33 themes (entities) in the co-design workshop session and 20 themes in the pilot evaluation session.

#### Usability Testing

For detecting usability issues, we applied a quantitative method by Sauro and Lewis [63]. We prioritized the usability issues according to their severity, considering the following factors: how much the issue has impacted the user trying to achieve the task, how critical the task was, and what was the frequency of the issue. Thus, the severity of issues was calculated by multiplying task criticality, impact, and frequency of occurrence. Overall, we detected 69 usability issues.

Each issue was then assigned one or more solutions. To choose the optimal solution to each usability issue, we considered 2 factors: complexity and effectiveness. Finally, a cost-benefit ratio (return on investment [ROI]) was calculated by dividing the effectiveness by the complexity. We decided which solutions to implement based on the following criteria: the solution had an ROI of 5 or more, was technically feasible, and was not solving the issue already covered by another solution with higher ROI. Overall, out of 56 conceptualized solutions, we arrived at 22 final solutions to be implemented. For a detailed description of the applied method, refer to Sauro and Lewis [63] and Rosemberg [75].

#### Heuristic Evaluation

Applying Dowding and Merrill’s [64] heuristic evaluation, we assessed 10 usability factors using 49 close-ended questions. Usability factors had varying question counts; the maximum score per factor was calculated by summing questions assigned to it. Scores obtained per expert were calculated by summing the questions answered yes by each expert, per factor. A mean score was calculated by summing individual expert scores and dividing by the number of experts. The final result was the mean score divided by the maximum score (per factor and cumulatively). Next, overall severity rankings per usability factor were assigned, ranging from 0 (no usability problem) to 5 (usability catastrophe). Finally, qualitative analysis of comments per factor informed suggestions on future implementations. For a detailed description of the applied method, refer to Dowding and Merrill [64] and Nielsen [71].

### Ethical Considerations

Informed consent was obtained from all participants prior to inclusion. Participation was voluntary, and participants were informed that they could opt out at any time. They were provided with information on how their data would be processed and that study data would be retained for no longer than 2 years from study start. No ethics or institutional review board approval was required for this work.

According to the Swedish Ethical Review Act (2003:460) [76], this study did not require formal ethical approval because it did not involve sensitive personal data, biological material, or any physical or psychological intervention. Only non-sensitive demographic information (country of residence, age, gender, profession) was collected. All participants were adults and data were pseudonymised using participant ID codes prior to analysis. Participants also provided consent for publication of the study results.

## Results

### Diagnosis Phase: DPs and Low-Fidelity Prototype

We have implemented DPs into a v1-low-fidelity prototype in PowerPoint. One dashboard element could be assigned to multiple DPs, but each assignment involved different functionalities of the element. Some DPs were action-oriented (DP1a and DP3a), and all material properties were assigned to them. We show how DPs shaped the v1-low-fidelity PowerPoint prototype in Table 3.

**Figure 4.**
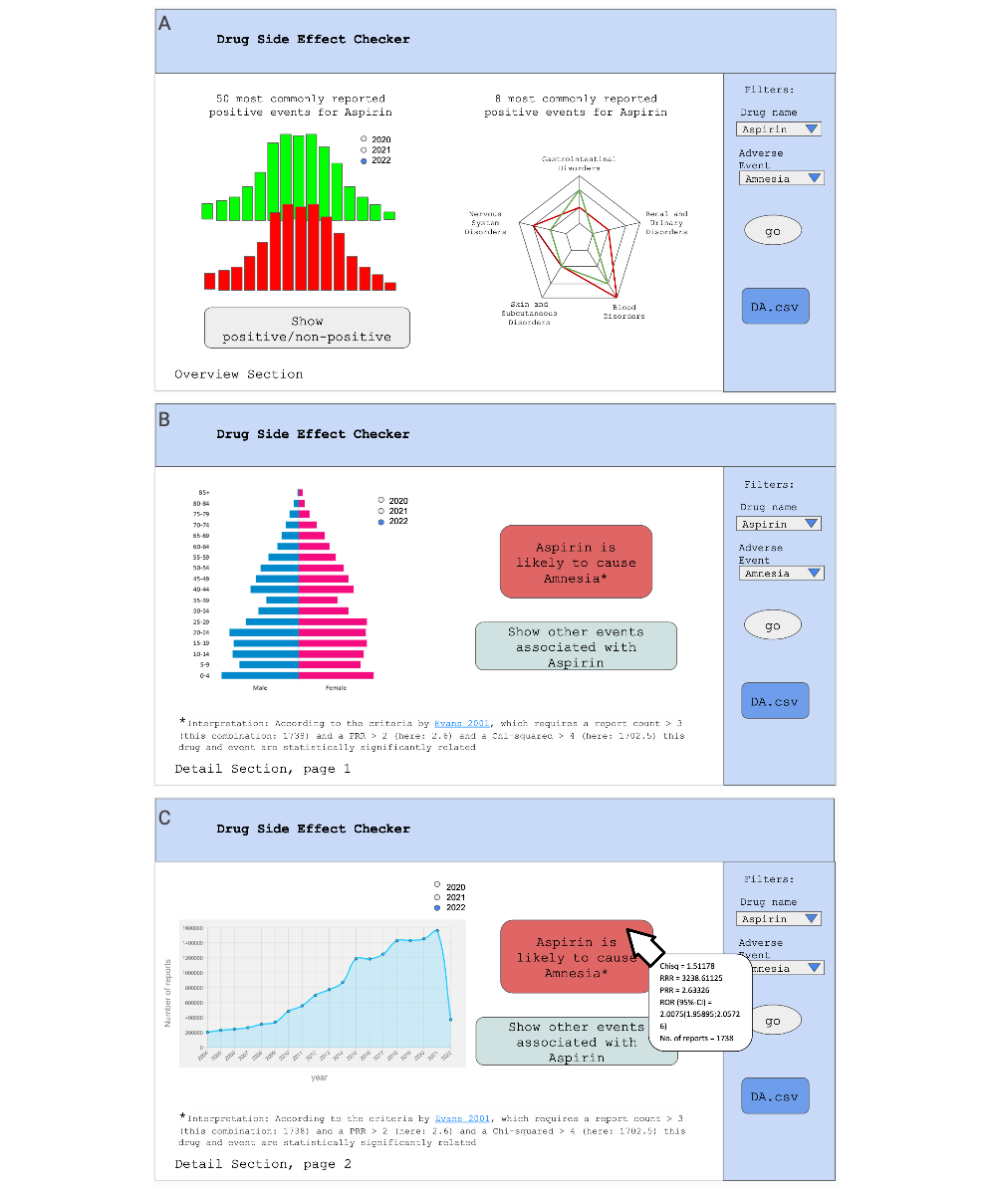
An excerpt from the v1-low-fidelity PowerPoint prototype. (A) The overview section. (B-C) The detail section.

To afford exploration of data of varied granularity (DP2b), we created two dashboard sections: (1) an overview of common side effects for the chosen drug (the overview section) and (2) a detailed analysis of specific drug-side effect pairs (the detail section), split in separate pages where different visualization techniques were used (eg, a graph with age-gender distribution of affected populations). Additionally, we added drug and side effect search fields (Figure 4A–C).

To afford identification of patterns and trends (DP1b), the overview section displays a histogram of commonly reported side effects for a queried drug. Users could toggle between positive and negative side effects using buttons (Figure 4A). To afford monitoring the progression of relevant states (DP1c), a line graph with a year filter tracked reports for drug-side effect pairs over time (Figure 4C). Additionally, to afford improved attention management (DP2c), key graphs were positioned top-left, and warning messages were color-coded.

The system provided downloadable evaluation files containing detailed drug safety metrics (DA.csv, Figure 4A–C) to afford evaluating alternatives of actions (DP3b). Warning messages indicated whether an event was statistically significantly related to queried side effects and provided DPA information on mouse hover (Figure 4C). Two affordances, obtaining immediate practical benefits while using the dashboard (DP1a) and active involvement in exploring the data (DP3a) were expected to be enacted across all prototype features, as they aligned with the prototype’s purpose.

**Table 3.** Instantiation of material properties.

Design principle	Instantiation of prototype features
DP1a	Initiated by all features and iteratively refined in response to users’ input
DP1b	Graphs belonging to the overview section, warning messages with DPA^a^ metrics on hover
DP1c	Line graph with the number of reports per drug-ADR^b^ pairs, with year filters
DP2a	Interactivity of the graphs (eg, buttons to toggle between positive and negative ADRs), with year filters
DP2b	Splitting the dashboard into the overview and detail sections, with year filters
DP2c	Placing the graphs on the top-left, color-coded warning messages
DP3a	Initiated by all features expected to elicit values related to the transparency of drug safety data or the user engagement in health information tracking
DP3b	DPA metrics as downloadable files (DA.csv), warning messages with DPA information on hover

a DPA: disproportionality analysis.

b ADR: adverse drug reaction.

### Diagnosis Phase: Requirements Gathering

Through a collaboration with a group of experts in a co-design workshop, we gathered the requirements for the prototype development. The v1-low-fidelity PowerPoint prototype was used in this session to visualize content of the dashboard, serving as a canvas for design activities. We summarize the principal findings in Table 4.

**Table 4.** Principal findings from the co-design workshop.

Feature or element	Related DPs^a^	Feedback
Indication graph	DP2b (overview and details) DP3b (decision-making support)	Offers details on common prescriptions for a queried drug, adding an extra variable to the displayed dataset.
Scrolling	DP2c (attention stimulation)	Use scrolling instead of pagination, it is a preferred way for users to interact with the dashboards.
Drug and event search fields	DP2b (overview and details)	Users seek information on potential side effects both before and after drug administration. Separate search fields allow users to look up a drug only (before an event occurs) or a drug and ADR^b^ (after an event occurs).
DPA^c^ metrics files	DP3b (decision-making support) DP2b (overview and details)	To influence decision-making of professional users, DPA files should include formulas and information on statistical models used for calculations, as well as the interpretation of calculated indicators.
Removing DPA info from the warning message’s hover	DP2b (overview and details)	While beneficial for researchers, high-level granularity data may confuse nonprofessional users. Access to DPA metrics is provided through downloadable files.
Age-gender distribution graph with modified age ranges, positioned top-left	DP2b (overview and details) DP3b (decision-making support) DP2c (attention stimulation)	The graph provides information on common demographic attributes (age and gender). Using 10-year ranges provides optimal data granularity.
Histogram of most commonly reported side effects, positioned top-left	DP2b (overview and details) DP2c (attention stimulation) DP2a (customized views) DP1b (causality between variables)	The graph provides an overview of the most commonly reported ADRs, with the option to customize the display using a histogram filter (Related, Unrelated, All events). Limiting the bars to 20 provides optimal data granularity.
Per drug, there is a line graph showing the sum of side effects reported over time (the first line graph) and a second line graph illustrating specific side effects reported over time	DP1c (state-tracking) DP2a (customized views)	For researchers, viewing time series of drug-ADR report numbers is crucial. The second line graph helps identify, for example, specific events contributing to sudden increases in ADRs in selected years. The histogram filter is linked with line graphs, enhancing customization options.

a DP: design principle.

b ADR: adverse drug reaction.

c DPA: disproportionality analysis.

#### Adjusting Data Granularity

**Figure 5.**
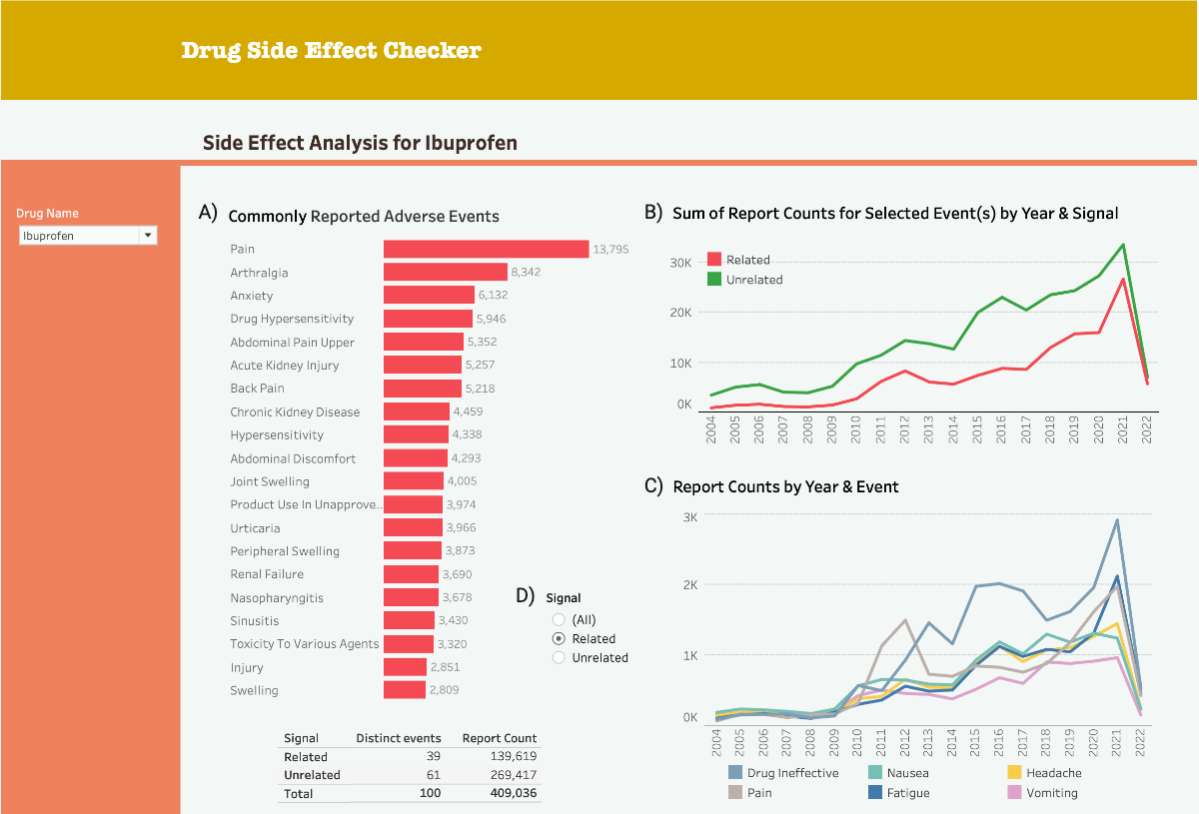
The first Tableau (v2-high-fidelity) prototype with analysis for ibuprofen (the overview section). (A) Modified histogram. (B) The first line graph. (C) The second line graph. (D) The histogram’s filter.

**Figure 6.**
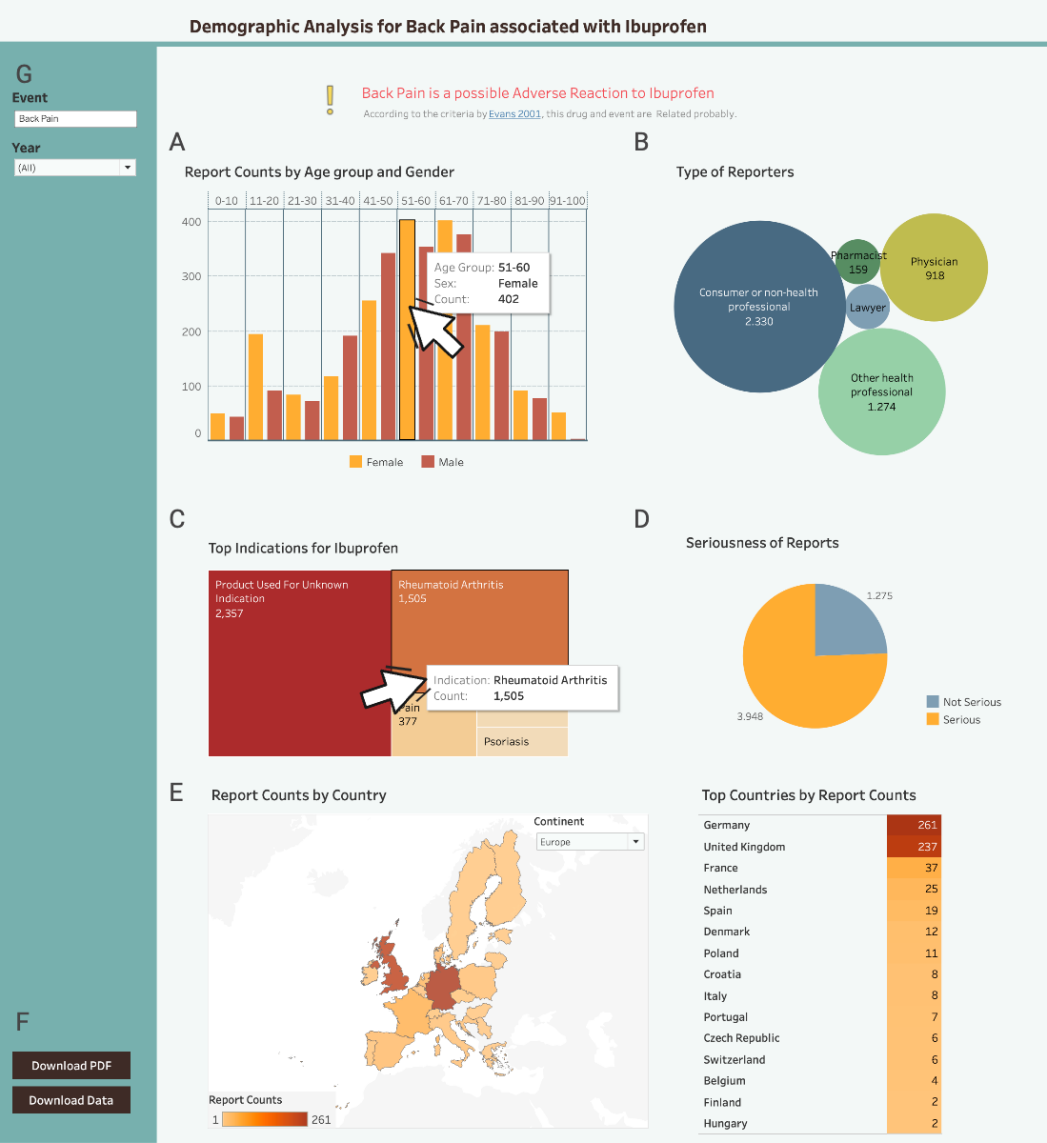
The first Tableau (v2-high-fidelity) prototype with analysis for ibuprofen and back pain (the detail section). (A) Modified age-gender distribution graph. (B) The reporter’s type of occupation. (C) The indication graph. (D) The seriousness of the outcome. (E) The map with number of reports per country and the associated table. (F) Download buttons. (G) The event search field.

We found evidence that the dashboard supported the exploration of data of varied granularity (DP2b). Feedback from experts suggested adjusting the detail level on display. Some elements were considered too detailed, resulting in information overload, especially on the histogram and the age-gender distribution graph. Recommendations included fixing the histogram’s displayed bars at 20 and setting age intervals on the age-gender distribution graph to 10 years (Figures 5A and 6A, respectively). Additionally, it was suggested that displaying detailed DPA metrics while hovering the warning message might confuse users unfamiliar with similar statistical methods, as per E1: this proportional reporting ratio and all that (...) Not super easy to interpret, right? This is greater than 2, but like, what is the upper bound? What is the lower bound? Like, how low can it go? (...) These values are not so easy to understand if you’re not the professional who uses them.

Thus, the DPA information was removed from the warning message and added to downloadable DPA files, together with formulas for score calculation and interpretation of indicators. This was also in line with the suggested increase of data granularity on some occasions. Likewise, it has been proposed to implement a functionality to view the data by the condition for which a drug was prescribed. Finally, while initially considered, the BMI variable was ultimately excluded from the age-gender distribution graph. Although useful for analysis, BMI is rarely provided in the reports. Therefore, it was necessary to adjust the intended data granularity to what was available in the datasets. Considering all the above, we revised the following DP: DP2b: It provides an overview and details, while controlling overview-to-detail ratio, so that the system affords exploring data of different granularity levels in drug safety surveillance.

Accounting for different data granularity interest among various user groups, and considering that customizable level of detail in displayed data can reduce distraction and potentially influence decision-making, we revised another DP: DP3b: It provides features to support decision-making and cluster them by the needs of distinct user groups, so that the system affords users to evaluate what-if scenarios and consider different alternatives in drug safety surveillance.

#### Scrolling Over Pagination

Feedback suggested changing from pagination to scrolling, as users often prefer to scroll, rather than having to navigate through multiple pages. Pagination is also considered more expensive in terms of browsing and might significantly slow the system down. Thus, we revised the DP: DP2c: it provides features that stimulate attention, place important elements on the top-left area of the dashboard, and promote scrolling over pagination, so that the system affords improved attention management while using the dashboard in drug safety surveillance.

### Iteration 1

#### Design and Development: First Tableau Prototype

We transformed the v1-low-fidelity PowerPoint prototype into a v2-high-fidelity dashboard in Tableau, incorporating refined DPs based on the guidance from the co-design workshop. Figures 5 and 6 show the prototype after the first iteration.

After requirements analysis, to better afford monitoring the progression of relevant states (DP1c), we introduced 2 graphs: a line graph showing all side effect reports per drug per year (Figure 5B) and a line graph showing specific side effect reports per drug per year (Figure 5C).

For exploration of data of varied granularity (DP2b), we added an indication graph showing the conditions for which the queried drug was prescribed when a side effect occurred (Figure 6C). In line with revised DP2b, we modified the age-gender distribution graph (Figure 6A). We also modified the histogram by reducing the number of bars to better afford identification of patterns and trends (DP1b). A map displayed side effect distribution per country with interactive continent selection and year filtering (Figure 6E).

With regard to improved attention management (DP2c), we positioned the histogram and the age-gender distribution graph on the top-left overview and detail sections, respectively. We replaced pagination with scrolling, aligning with DP2c. To better afford evaluating alternatives of actions (DP3b), we introduced safety signal reports with DPA information, displayed upon clicking the download data button (Figure S1 in Multimedia Appendix 3). To better afford exploring data from different angles (DP2a), all graphs in the overview section were made interactive: for example, selecting up to 6 histogram bars was reflected in the second line chart, where up to 6 individual events were displayed. It was also possible to limit the dataset by year ranges (Figure S2 in Multimedia Appendix 3).

#### Demonstration and Evaluation: Pilot Evaluation

While conducting a pilot evaluation, we were looking for suggestions on possible improvements to the prototype before embarking on usability testing. Overall, we received advice to implement a getting started tutorial containing screenshots of the dashboard elements with a short explanation on salient functionalities (eg, how to remove the selection and how to select using keyboard or mouse).

Importantly, expert E3 suggested implementing a feature related to selection-keeping—when the user chooses one or more side effects by clicking on the histogram bars, and then changes their drug selection, the dashboard should keep the selection of side effects. This feature can be useful to quickly compare drugs from the same drug classes (eg, ibuprofen and aspirin). According to E3: if you want to compare that one drug to another drug, to see if it (selected side effect) also occurs really often, then this makes sense (...) And you can see it (selected side effect) directly in the ranking.

This feature was in line with DP3b, potentially enhancing the decision-making of professional users.

### Iteration 2

#### Design and Development: Updating Prototype Features

**Figure 7.**
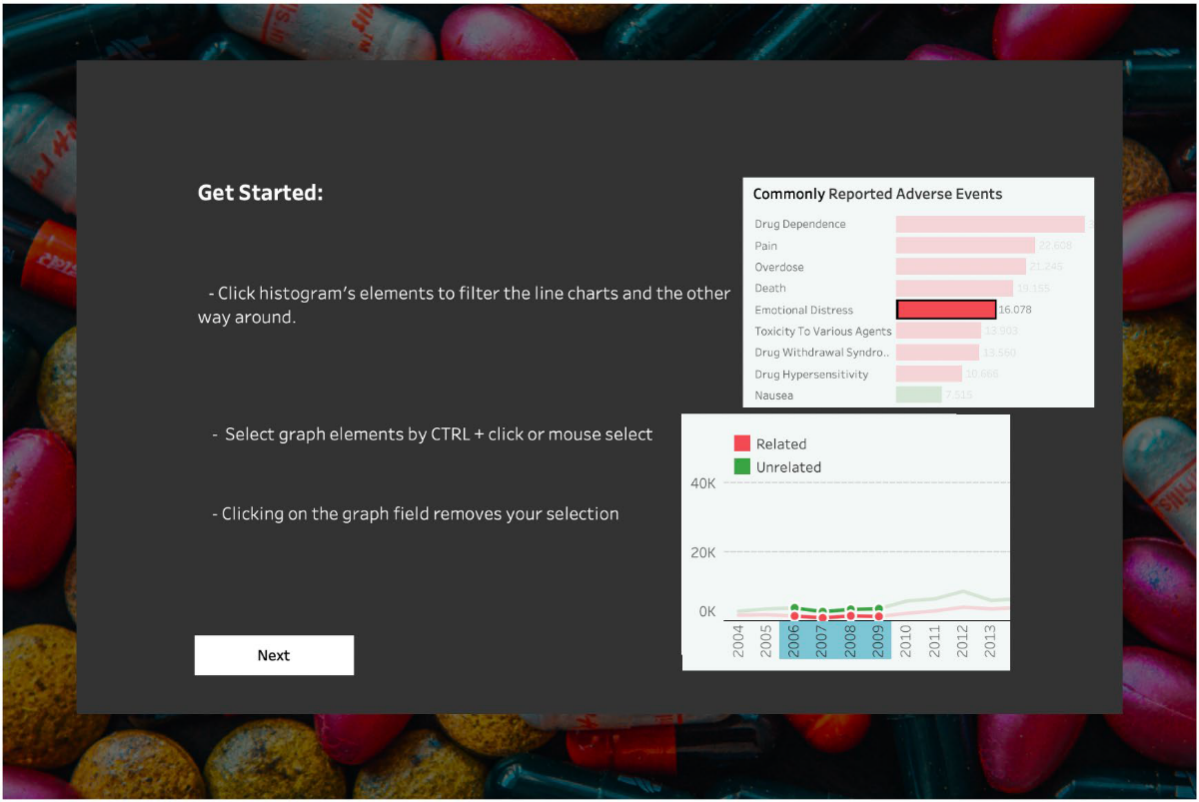
An exemplary page from the getting started tutorial in the v3-updated prototype.

We updated the system by implementing selected findings from the pilot evaluation session. Thus, we introduced a getting started tutorial with informative screenshots that guided users through the dashboard functionalities (Figure 7).

To better afford evaluating alternatives of actions (DP3b), we introduced the selection-keeping feature. For a detailed explanation, refer to Figure S3 in Multimedia Appendix 3, where we present a real-world scenario demonstrating the comparison of 2 commonly used pain-relieving drugs: ibuprofen and acetaminophen (also known as paracetamol).

#### Demonstration and Evaluation: Usability Testing

We conducted usability tests with nonprofessional users (refer to Multimedia Appendix 4 for details). We found several usability issues that could hinder obtaining immediate practical benefits while using the dashboard (DP1a). We grouped the issues according to the affected dashboard features or elements, and we discussed them in the corresponding order.

The getting started tutorial was hard to follow and long. Users struggled to grasp how the individual screenshots relate to the dashboard concepts. This confusion arose because the screenshots presented isolated dashboard elements instead of the dashboard as a whole. The inability to skip or revisit the tutorial was also a major issue. Regarding the histogram and associated filter**,** users had trouble understanding how to select bars by clicking or by dragging sections. Finding the histogram’s filter (Figure 5D) took more than 15 seconds, which could hinder identification of patterns and trends (DP1b), as failing to use the filter could impede the thorough exploration of data.

Usability problems also arose for year selection across different dashboard sections. Users didn’t fully explore year selection options and found deselecting elements challenging (Multimedia Appendix 4). The absence of a year selection field in the overview section could impede exploration of data of varied granularity (DP2b) and, in consequence, interfere with monitoring the progression of relevant states (DP1c).

Some dashboard elements lacked visibility and clear explanations of their utility. For example, the event search field was not visible enough (Figure 6G) and the utility of the second line graph was not clear to the users. Additionally, the dashboard language was too technical. We also found issues with download buttons, DPA metrics files, and safety signal reports. Multiple download buttons and files made downloads difficult. Moreover, the download dialog box was located beyond eye reach and the system failed to inform about the download status. Finally, safety signal reports were too detailed and specific. According to C2, the report provided “unnecessary information overload” in which the participant was not interested.

End questions revealed users’ confusion about the data source and the meaning behind the evaluation of side effects as related or unrelated to a drug by data mining algorithms. This lack of understanding could constrain evaluating alternatives of actions (DP3b). However, users found the system attractive for exploring drug safety information (DP3b). They were often encouraged by its interactivity to explore data from different angles (DP2a) and expressed their interest in using the system as an auxiliary drug information source. C4 suggested that people generally search online for drug safety information, seeking medical advice on websites of compromised quality. The participant considered the dashboard a suitable information source even for users with no pharmacovigilance background, as long as it is supplementary to professional medical advice. Similarly, C3 suggested the system could be useful for direct comparison of drugs with the same active substance produced by different manufacturers. C3 often asks for a cheaper drug alternative at the pharmacy and could use the system to compare the safety information of equivalent drugs.

### Iteration 3

#### Design and Development: Updating Prototype Features

##### Introducing the Video Tutorial

To better afford obtaining immediate practical benefits while using the dashboard (DP1a), we modified the tutorial. Usability testing revealed that users learn best by doing, but they require basic knowledge to get started. Well-designed tutorials allow users to uncover the true potential of the app and understand the associated benefits [77]. Following the idea that undiscovered features practically do not exist, we replaced the tutorial with the animated walkthrough video.

**Figure 8.**
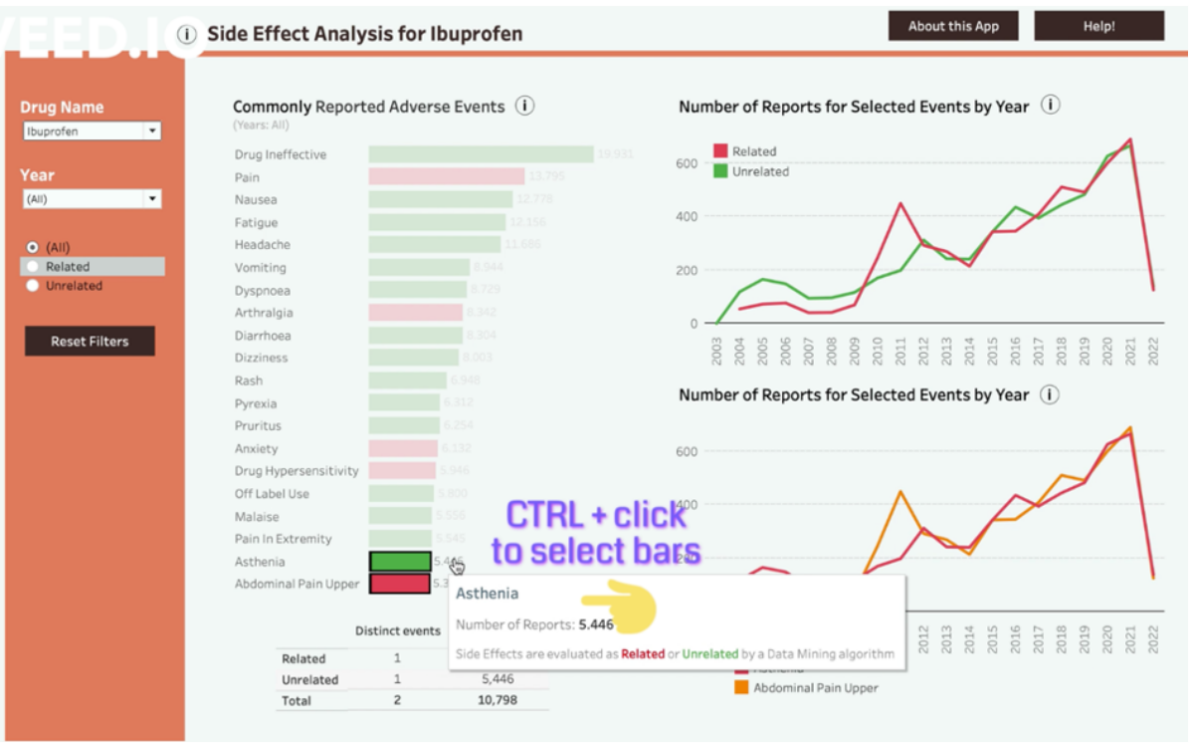
A frame from the getting started video tutorial of the v4-updated prototype.

We recorded key features in a brief video (more than 1 minute) with subtitles explaining dashboard actions. We used icons (eg, pointing finger and large mouse cursor) to guide users and added a skip video button for more experienced users (Figure S5 in Multimedia Appendix 3). Finally, we included a help button allowing users to rewatch the tutorial (Figure 8).

##### Emphasizing the Utility of Dashboard Elements

**Figure 9.**
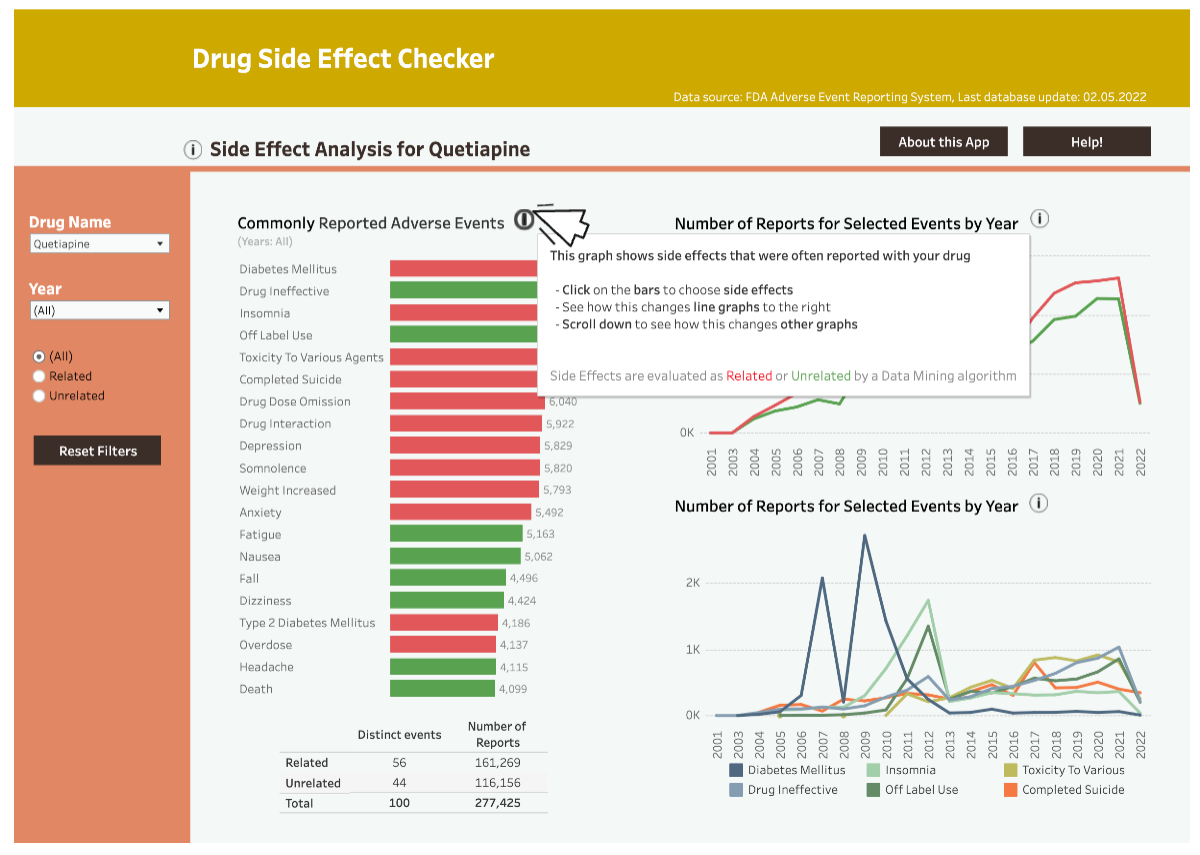
Histogram with the info icon. Prototype v4.

We addressed many usability issues by adding short labels to dashboard elements (Figures9  10).

To better afford monitoring the progression of relevant states (DP1c), we included an info icon next to graphs and section headers. Users could see information about graph functionality and displayed data, as well as the explanation of complex terms. We also included information on the content of the overview and detail sections and explained how graphs interacted with each other.

Similarly, we expected the issue of insufficient visibility and unclear utility of some elements (eg, the event search field and the second line graph) would be addressed by including additional information in the tutorial (Figures S6 and S7 in Multimedia Appendix 3). Finally, we implemented the “About this App” section (Figure 11), where users could find information about the data source, safety signal evaluation, possible limitations to the tool, and external links.

Considering all the above, we refined the DPs: (1) DP1a: it provides features to address the bootstrap problem, add walkthrough videos, and explain dashboard elements**,** so that the system affords immediate use of the dashboard in drug safety surveillance. (2) DP1c: it provides features to track changes in the variables and highlight their utility, so that the system affords tracking of the progression of relevant states in drug safety surveillance.

**Figure 10.**
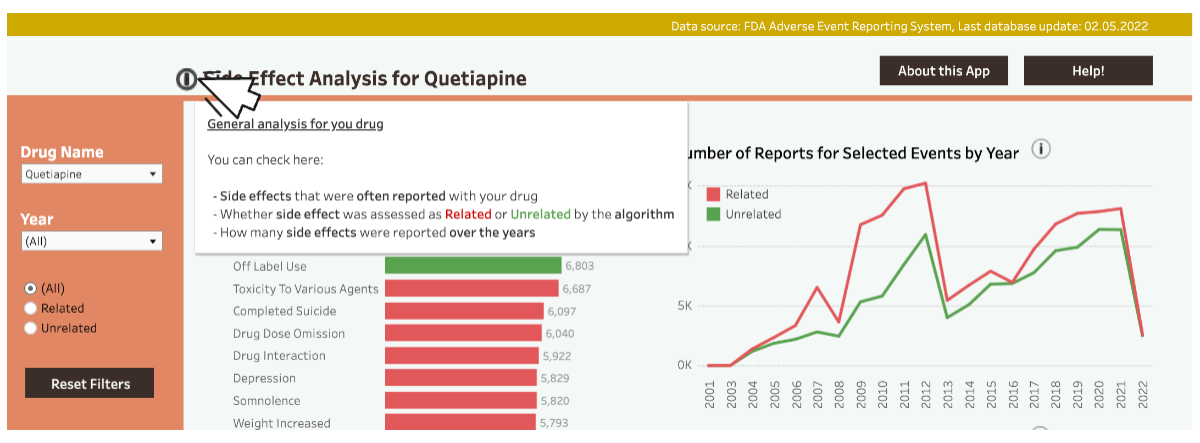
The overview section with the info icon. Prototype v4.

**Figure 11.**
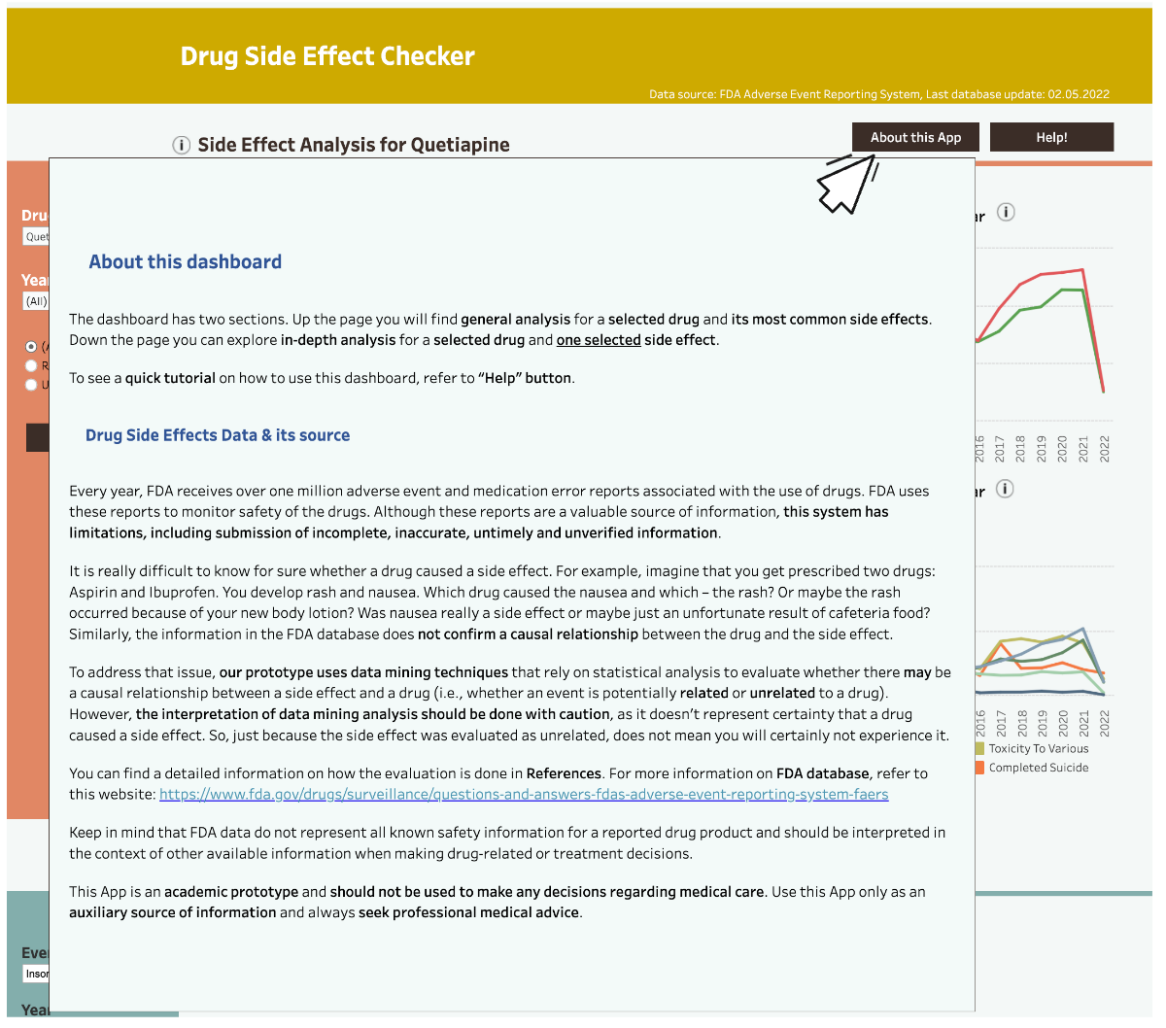
The About this App dashboard section. Prototype v4.

##### Signalizing the Uncertainty of Data Mining Results

To better afford identification of patterns and trends (DP1b), we added the information on the uncertainty behind DPA evaluation and advised caution when interpreting the results. Since safety signal detection can be used for hypothesis building, but not testing, we found it crucial to highlight these concepts. Safety signal detection is a starting point for further investigations, not a conclusion. Considering ethical and safety concerns, appropriate warnings are necessary.

**Figure 12.**
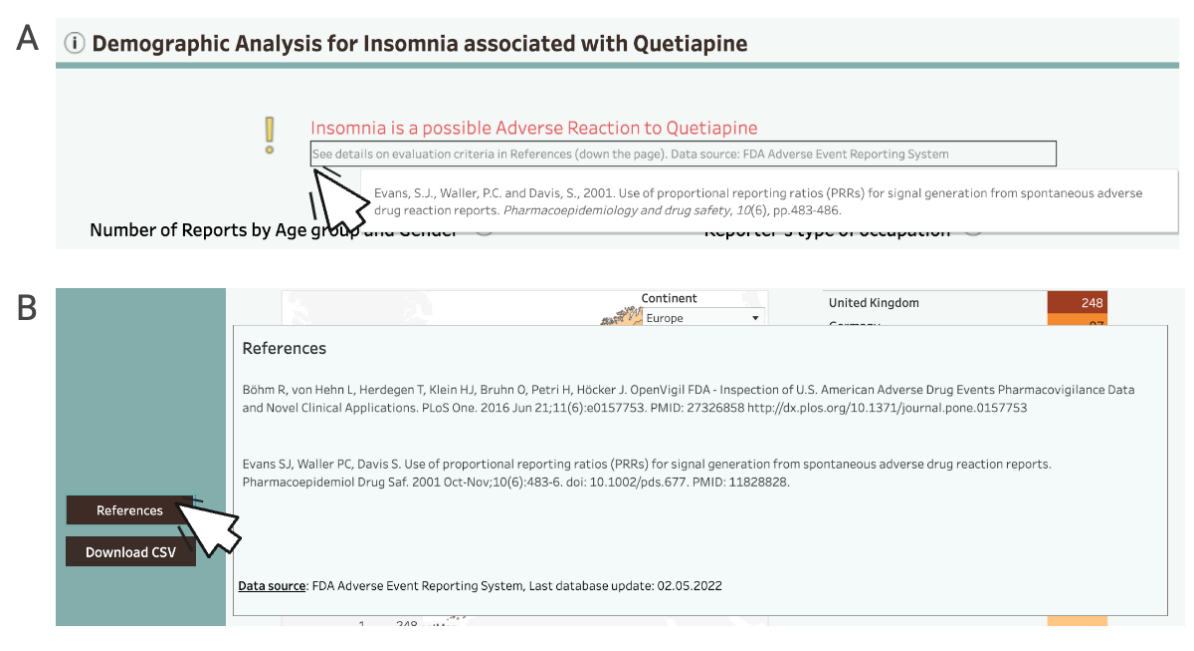
(A) The warning message with the information on hover. (B) The References button with the information on hover. Prototype v4.

We addressed all the above in several ways. First, we provided the explanation on the interpretation of DPA in a disclaimer (not shown) and in the About this App section. Accounting for users less familiar with the topic, we provided an illustrative real-world case scenario to explain the concepts. We also implemented the References button, providing the links to evaluation criteria and including similar information in the warning message (Figure 12). Finally, we added links to external resources on many occasions and further enriched DPA files in explanation. In line with the above, we refined the following DP: DP1b: it provides features to describe possible causality between variables and signalize uncertainty behind DPA results in different ways, so that the system affords identification of patterns and trends in drug safety surveillance.

Together, these changes replace specialist terminology with plain-language labels and confine full statistical detail to downloadable files, reducing cognitive load for lay users while preserving transparency for professional review.

##### Improving the Usability of Other Elements

**Figure 13.**
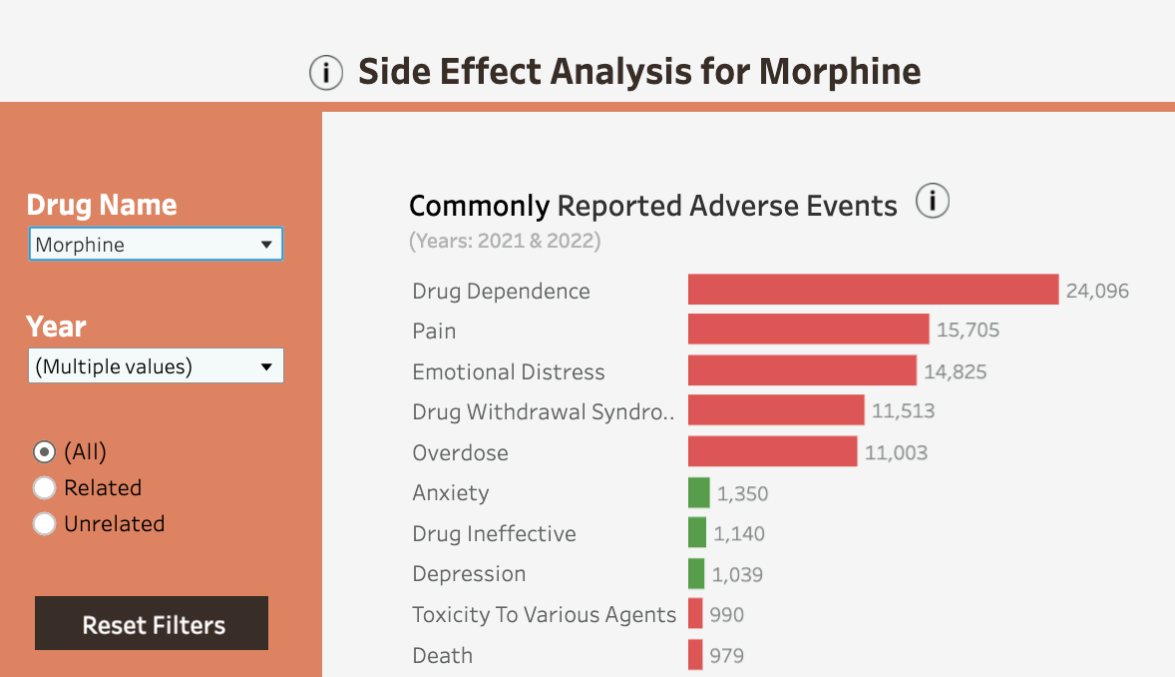
Histogram with the side effect filter (All, Related, Unrelated), year filter, the Reset Filters button (bottom left), and information on current year selection (in fine print, gray). Prototype v4.

To address the usability issues related to the histogram, and to improve attention management while using the dashboard (DP2c), we relocated the histogram’s filter to the upper-left corner, removed the confusing filter’s name, and added a histogram’s year filter with the information about the current year selection. We also implemented a Reset Filters button to unselect elements in the overview section (Figure 13).

To facilitate obtaining immediate practical benefits while using the dashboard (DP1a), we simplified the language and modified download options, removing redundant download buttons and files. With regard to evaluating alternatives of actions (DP3b), and considering different data granularity needs of users (DP2b), we removed safety signal reports from the background of the download page and simplified the view. Finally, we moved the download dialog box to the eye reach (Figure S8 in Multimedia Appendix 3).

The summary of all solutions implemented after usability testing is shown in Table S2 in Multimedia Appendix 4. The final version (v4) of the prototype was made available online [78]. We recommend using Google Chrome as a browser for a better experience.

### Demonstration and Evaluation: Heuristic Evaluation

**Figure 14.**
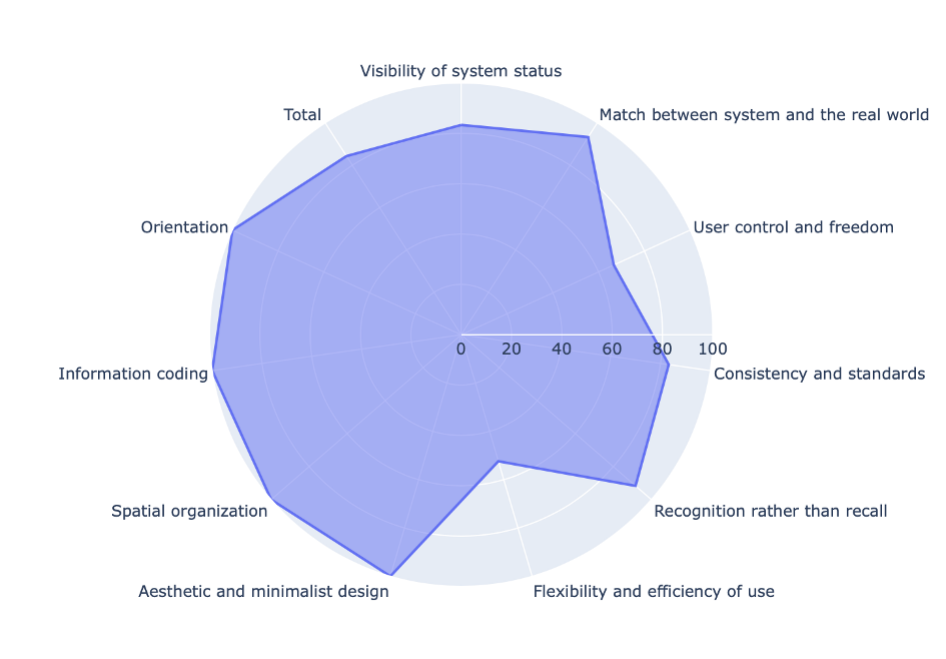
Usability scores per usability factor. Total is the overall usability score.

We concluded the DSR cycle by performing heuristic evaluation (Figure 14). We identified 4 out of 10 usability factors with the lowest usability score: flexibility and efficiency of use (52%), user control and freedom (67%), visibility of system status (83%), and consistency and standards (83%). Two usability factors had the usability scores above 90%: recognition rather than recall (92%) and match between system and real world (93%), while 4 usability factors had the usability scores of 100%: aesthetic and minimalist design, orientation, spatial organization, and information coding, where the last 3 factors were related to dashboard visualization. The overall usability score was 84% (Table 5).

**Table 5.** A summary of usability factors with corresponding usability scores. Maximum score is a sum of close-ended questions per usability factor (eg, usability factor #9 had 2 questions, thus its maximum score was 2). E1, E2, and E3 are the scores assigned to each usability factor by each expert (ie, the sum of questions answered as yes by E1, E2, and E3). The result is the mean score divided by the maximum score, displayed as a percentage. Total is the overall usability score.

ID	Usability factor	Maximum score	E1	E2	E3	Mean score	Result (%)
1	Visibility of system status	6	5	5	5	5	83
2	Match between system and the real world	5	5	5	4	4.7	93
3	User control and freedom	5	4	3	3	3.3	67
4	Consistency and standards	6	5	4	6	5	83
5	Recognition rather than recall	4	4	3	4	3.7	92
6	Flexibility and efficiency of use	7	4	4	3	3.7	52
7	Aesthetic and minimalist design	7	7	7	7	7	100
8	Spatial organization	3	3	3	3	3	100
9	Information coding	2	2	2	2	2	100
10	Orientation	4	4	4	4	4	100
	Total	49	43	40	41	41	84

The overall severity ratings (Table 6) were in line with the obtained usability scores in most cases. Usability factor #3—user control and freedom was assigned the severity rating of 2 (minor usability problem), while the usability factor #1—visibility of system status was assigned the severity rating of 1 (cosmetic problem only) by all experts. Surprisingly, the usability factor #6—flexibility and efficiency of use, although it obtained the lowest overall usability score, was assigned the severity rating of 0 (no usability problem) by all experts. Several other usability factors were assigned the severity rating of 1 by some experts: usability factor #2—match between system and the real world (E3), usability factor #4—consistency and standards (E1), and usability factor #5—recognition rather than recall (E2). All remaining usability factors were evaluated as 0 (no usability problem).

The majority of usability issues referred to the flexibility and efficiency of use. We found evidence that the system lacked the ability to control display configurations and to hide unnecessary displays. Interestingly, although all participants noticed the lack of the option to hide unnecessary displays, it was not considered essential. According to E1: no buttons to hide or unhide panels, but not sure if the button is necessary. Information is well spread.

This could explain why this usability factor—although it obtained a relatively low usability score—was assigned an overall severity rating of 0 (no usability problem). Yet, as we saw a clear consensus regarding insufficient control of display configurations, we refined the following DP: DP2a: it provides features to support customized views and to control display configurations, so that the system affords examining different aspects of data in drug safety surveillance.

**Table 6.** Overall severity ratings assigned by experts (E1, E2, and E3) in heuristic evaluation.^a^

ID	Usability factor	Overall severity rating		
		E1	E2	E3
1	Visibility of system status	1	1	1
2	Match between system and the real world	0	0	1
3	User control and freedom	2	2	2
4	Consistency and standards	1	0	0
5	Recognition rather than recall	0	1	0
6	Flexibility and efficiency of use	0	0	0
7	Aesthetic and minimalist design	0	0	0
8	Spatial organization	0	0	0
9	Information coding	0	0	0
10	Orientation	0	0	0

a A score of 2: minor usability problem, 1: cosmetic problem only, 0: no usability problem.

Feedback also suggested that the system lacked clear exit options and a universal undo function, hindering user control and freedom. We also found support for the utility of the reset button; however, some participants noted that it did not deselect all elements when clicked. Regarding the visibility of system status**,** participants pointed out insufficient system feedback on its status. E3 identified a lack of visual feedback when selecting elements on some graphs. Although this action affected histograms, it was not communicated to the user. Similarly, E2 identified missing information on year selection in the detail section. Finally, concerning the consistency and standards**,** participants noted the absence of visual cues to identify active screens. E2 struggled to identify active screens when navigating between the main and download pages.

As a future agenda, we recommend implementing enhanced user control over display configurations, options to hide unnecessary displays, and adding a back or undo button. The functionality of the reset filters button should be improved to uniformly remove selections. Better feedback on the system status, like displaying selected years in the detail section, is crucial. Prompts and visual cues (eg, the event search box) should be placed where the eye is likely to be looking. Finally, all interactive elements (eg, the histogram and the first line graph) should provide feedback upon selection.

## Discussion

### DPs and the Value of Affordance Theory in This Study

Following the DSR methodology, we were able to empirically test and refine the initial, theory-driven DPs, by iteratively juxtaposing the results of experimental prototyping with theory-grounded, conceptual design recommendations. Consequently, we prove that the iterative translation of evaluation outcomes into prototype features results in refined design guidelines that could lead other practitioners in similar domains.

Throughout the study, we applied the affordance lens pragmatically: target affordances remained stable while prototype features were iteratively refined to instantiate them, which is in line with the view of Iivari [79] and Chandra et al [53]. Table 7 summarizes each principle in terms of user activity (affordance), enabling features (material properties), and the boundary conditions.

**Table 7.** The final list of refined design principles.

Design principle	Revised content
DP1a	Provide features to address the bootstrap problem, add walkthrough videos and explain dashboard elements, so that the system affords immediate use of the dashboard in drug safety surveillance
DP1b	Provide features to describe possible causality between variables and signalize uncertainty behind DPA^a^ results in different ways, so that the system affords identification of patterns and trends in drug safety surveillance
DP1c	Provide features to track changes in the variables and highlight their utility, so that the system affords tracking of the progression of relevant states in drug safety surveillance
DP2a	Provide features to support customized views and to control display configurations, so that the system affords examining different aspects of data in drug safety surveillance
DP2b	Provide overview and details, while controlling overview-to-detail ratio, so that the system affords exploring data of different granularity levels in drug safety surveillance
DP2c	Provide features that stimulate attention, place important elements on the top-left area of the dashboard, and promote scrolling over pagination, so that the system affords improved attention management while using the dashboard in drug safety surveillance
DP3a	Provide features to create public values, so that the system affords users’ engagement in dashboard use in drug safety surveillance
DP3b	Provide features to support decision-making and cluster them by the needs of distinct user groups, so that the system affords users to evaluate what-if scenarios and consider different alternatives in drug safety surveillance

a DPA: disproportionality analysis.

### Addressing Existing Research Gaps

Our literature review identified pharmacovigilance systems that did not sufficiently incorporate user requirements, with resulting usability shortcomings [17,18,32]. We respond to this by using the DSR as a methodology. Additionally, this research gap was closely related to the bootstrap problem and DP1a (designing for direct use). We tackled the bootstrap problem in 2 ways: first, by including the requirements gathering phase in the design cycle, we increased the probability of addressing the real needs of users and therefore solving the right problem; second, by giving the users the opportunity to engage with the prototype in the initial stages of development, we initiated its use from the early versions. This positively affects the quality of the prototype and increases the chances of its successful adoption in the future. By iteratively responding to users’ needs and targeting usability issues, we built a high-fidelity prototype that is usable for the evaluated tasks and user groups (overall usability score: 84%) and is designed to support sustained use; broader deployment remains future work.

We previously identified the lack of data mining techniques as a limitation of some of the systems for pharmacovigilance [17,21-23]. We suggested this was an important research gap to address, related directly to DP1b (causality between variables). We implemented DPA evaluation in the dashboard by including the information on potential safety signals [80]. We emphasized the uncertainty behind these results in multiple ways to make sure users were aware of their interpretation. Finally, the advantage of our prototype comes with improved visualization techniques. To that end, we followed the guidelines for visual dashboards by Bremser and Wagner [61] when deciding on visualization elements and features.

Consistent with DP3b (“cluster features by the needs of distinct user groups”), we view clinicians and pharmacists, researchers, and public users as distinct personas requiring layered functionality and language; our present DSR cycles cover the latter 2 groups (public users and research professionals), and clinician-facing tailoring should be undertaken in subsequent evaluations by others. In addition, regulatory decision-makers, policy analysts, and global health agencies represent further personas aligned with DP3b; though not evaluated here, they are recognized within our stakeholder scope and remain outside this study’s boundary conditions.

### Creating Public Value

Here, we articulate how the DPs and their instantiation can create public value when scaled and deployed in appropriate contexts; in this study, we assess feasibility and task-level usability rather than population-level impact.

We previously reviewed systems for pharmacovigilance that use proprietary databases or other data sources limited in diversity of stored information, volume, and availability [14-16,27,28]. To respond to this gap, related directly to DP3a (public value), we based the prototype on rich, open-access datasets with safety reports registered worldwide. In addition, both the code and the prototype are open-source and made available online, potentially contributing to the pharmacovigilance community. Moreover, through improved visualizations, we aim to enhance understanding and encourage users’ active participation in interpreting the information.

To counter the misinformation during the COVID-19 pandemic, Murthy [81] recommends addressing information deficits where there is a high public interest but limited quality information available. Additionally, the author advocates for the modernization of public health communications by developing new, credible, and evidence-based online tools to effectively convey information to communities. By providing transparent and accurate drug safety data from reliable sources, our system may contribute to fighting misleading information and negative attitudes toward medication.

Our system is also a step forward in a higher degree of patient empowerment (the restoration of patients’ access to public health data) and patient participation (the ability of patients to receive drug safety information and act upon it). In line with our findings, drug consumers could use the prototype for direct comparison of drugs with the same active compound, but produced by different manufacturers. Generic drugs (drug equivalents), although chemically the same as brand-name drugs (drugs produced by the pharmaceutical company that discovered them, protected by the patent), are often negatively perceived by drug consumers [82-84]. This is due to misbeliefs about brand-name drugs being better medicines per se.

There is strong evidence that, through a variety of incentives, medical doctors are influenced by pharmaceutical companies on which drugs to prescribe [85-88]. Physicians often enter contractual relationships with vendors, the latter being mostly representatives of brand-name drug companies, rather than cheaper generic drugs [89]. We suggest that by having a tool to directly compare primary safety profiles of drugs and their alternatives at hand, doctors may be encouraged to make more ethical decisions on their choice of collaboration with pharmaceutical companies.

### Enhancing the Decision-Making

We integrated exportable DPA files into the prototype, considering them the main enabler of decision-making. When combined with data visualizations, this feature can significantly benefit pharmacovigilance professionals, including those affiliated with pharmaceutical industry or research institutes. These same affordances are pertinent to policy analysts and global health agencies, who require transparent, auditable summaries and cross-jurisdictional trend overviews. Detecting unusual or previously unknown drug-ADR associations is a first step in drug safety investigations [23,90]. Identified signals prompt further research, influencing actions such as product labelingchanges or potential drug withdrawal [91].

Regulatory bodies such as FDA, WHO, and EMA systematically monitor drug safety signals. Unusual associations undergo thorough scrutiny, involving literature reviews, clinical study revisions, and exploration of biochemical mechanisms. Results are compared with product information, and pharmaceutical companies are asked to provide relevant data, promoting higher industry accountability [91,92].

### Contributions

#### Theoretical Contribution

Our work contributes to the prescriptive knowledge [93] by providing a list of refined, empirically-driven DPs. We suggest that, by delineating DPs, our work puts forward general design recommendations in similar solution domains. These can be extrapolated to a class of similar problems concerned with data visualization in pharmacovigilance. We do not claim the DPs are completely generalizable to other settings. Instead, they are put forward with the ambition to lead research on similar problems in wider contexts.

To our knowledge, there is no previous research that uses DSR methodology in designing dashboards for pharmacovigilance. By engaging in the DSR cycle, we were able to define affordances for similar systems, contributing to better understanding of the problem domain. These were (1) prompting users to engage with the dashboard (enabled by improved usability and attention management), (2) aiding analysis of what-if scenarios to support decision-making processes, (3) presenting data from different angles and at multiple granularity levels to examine its different facets, (4) showing changes in relevant variables over time, and (5) creating public value through generated insights.

Finally, we contribute to DSR by providing insights into how different requirements gathering and evaluation methods can support the development of visualization dashboards. While no claims can be made about the validity of these findings to other systems, we hope the techniques proposed here can be a useful instrument for identifying design issues early in the prototype development.

#### Practical Contribution

The practical contribution of our study lies in the developed artifact, publicly available online. Our system can be used by researchers and practitioners in pharmacovigilance, upon refinements. The code is open-source [94] and others can repurpose or reuse the scripts as partial solutions or inspiration for their projects. Though we confined our prototype to 5 drugs and 500 secondary events, the code can be adapted to an infinite number of drugs or secondary events to suit others’ needs. For larger multisource deployments, the ETL and DPA steps can be executed in batches without altering the front end. Because the user interface consumes flat exports, the prototype can be reproduced in fully open-source dashboards or a custom front end.

The prototype is intended to demonstrate and operationalize the DPs. While usable for the studied tasks, broader public deployment requires the scale-up steps outlined in the next section. Any public-facing release by adopters would also require clear plain-language disclaimers and risk communication for lay users, basic accessibility compliance, and a documented support pathway; these deployment activities are outside the scope of this study.

Our project however can be considered a hybrid open-source considering our reliance on the Tableau, which is a proprietary software. Table 8 provides an accurate description of the hybrid nature of our project with its respective components.

Finally, we do not claim full generalizability. The DPs are intended as reusable, prescriptive heuristics for similar dashboards within the stated boundary conditions; adaptation to clinician and pharmacist-facing workflows and additional user groups should be iteratively refined in those settings.

**Table 8.** Open-source status of project components.

Component	Status	Implication
Data	Open source	Fully accessible and usable by anyone
Code	Open source	All transformation and analysis code (eg, Python or R scripts) is on GitHub under an Open Source Initiative–approved license, allowing others to reuse and modify it
Dashboard prototype	Proprietary/Open access	The dashboard itself is viewable and accessible to anyone via Tableau Public (making it “open access”), but its dependency on proprietary Tableau software means others cannot easily reproduce, fork, or host a modified version without their own Tableau software or an equivalent proprietary viewer

### Limitations and Future Research Agenda

This study has some limitations. First, drug safety surveillance can be viewed differently, leading to different affordances than those outlined here. Second, the DSR cycle could be extended to yet another phase. Iivari et al [95] proposes a framework for DPs evaluation in terms of their reusability: through the criteria of accessibility, importance, novelty, actability and effectiveness. Similarly, empirical testing of how revised DPs apply to other settings could be done through their repeated application in different boundary conditions [52]. Future research could address these issues to test the quality of the outcomes of this DSR project. Third, to support broader use, the prototype should be expanded to all FDA drugs and events via direct application programming interface calls [21].

Establishing readiness for broad public deployment of the prototype will require expanded datasets and multisite evaluations beyond the scope of this study. Moreover, including data from other databases (eg, EudraVigilance and VigiBase) would improve DPA evaluation, but requires (1) source-specific mapping into the common schema, (2) robust cross-repository deduplication, and (3) compliance with licensing and governance constraints. If further deployments are undertaken, evaluation can focus on user engagement, operational reliability, and decision support alignment with expert assessments, as these activities are beyond this study.

Relatedly, in the final prototype version, we keep shortcuts and advanced controls to a minimum to maintain clarity for nonprofessional users. This design choice explains the lower expert scores on flexibility, efficiency of use, and user control and freedom, which were nevertheless judged as low-severity issues. For expert-only deployments, future implementers can add more controls and configuration.

Importantly, our participant sample represented young and digitally proficient (late 20s to mid 40s), and several nonexpert participants held technical or professional roles (eg, data science, biomedical engineering, and humanitarian logistics). This sampling may limit generalizability to older adults and individuals with lower digital health literacy [96]. Older adults are more likely to experience polypharmacy [97] and may have distinct interaction needs (eg, larger text, higher contrast, simplified and error-tolerant interactions) [98 ], which we did not assess in this study. Future independent evaluations should specifically include older adults and lower-literacy users and consider accessibility guidance (eg, larger typographic scales, high-contrast palettes, and keyboard navigability) when adapting the dashboard for these groups.

Relatedly, some nonprofessional users found data mining terms challenging even when aided by tooltips and tutorials. The present prototype mitigates this by using plain-language labels, info icons, an “About this App” section, and by keeping advanced formulas in downloadable files; however, residual risk of misinterpretation remains. We therefore reiterate that DPA supports hypothesis generation rather than confirmation and should be interpreted cautiously by lay audiences. To reduce potential user anxiety, we emphasize this uncertainty in plain language and signpost users to seek professional advice for individual decisions. However, we did not evaluate affective outcomes (eg, worry and decisional conflict) among lay users; given the potential for spontaneous report dashboards to provoke anxiety, such outcomes should be assessed in independent evaluations by others.

Furthermore, it would be beneficial to implement a higher variety of data sources: scientific literature, clinical studies or data from text mining [20]. Also, the prototype would benefit from comprehensive benchmarking, such as head-to-head comparisons with existing pharmacovigilance tools on performance, cost, or user satisfaction. Future work could also expand DPA methods to improve the validity of detected safety signals. Integrating and systematically evaluating additional machine learning approaches for ADR signal detection could enhance analytical breadth and validate performance across settings.

Finally, integration into health care IIs (eg, electronic health records) was outside the scope of this DSR prototype. The artifact targets public FDA data and does not process identifiable patient records. Any operational deployment by adopters would require institutional governance (eg, data use agreements, access control, auditability, and security increase) and, where applicable, compliance with jurisdictional regulations. The use of standard terminologies, decoupled Python scripts, and portability to open source ( Multimedia Appendix 2) is intended to ease such translation, but we do not evaluate these deployment aspects here.

### Conclusion

In this study, we applied the DSR cycle to investigate the design of visual dashboards to support activities within pharmacovigilance. To that end, we identified required affordances, developed corresponding DPs that satisfied them, and demonstrated their implementation, showing that they brought about expected outcomes. The developed DPs are anticipated to be applicable in various pharmacovigilance settings. Finally, we hope our research will be a catalyst of further research in similar domains.

## Supplementary material

10.2196/75936 Multimedia Appendix 1 Demographics of the target groups.

10.2196/75936 Multimedia Appendix 2 Design science research approach details.

10.2196/75936 Multimedia Appendix 3 Prototype in Tableau.

10.2196/75936 Multimedia Appendix 4 Usability testing and an overview of solutions implemented.

## Abbreviations

ADR: adverse drug reaction
AI: artificial intelligence
DiP: dashboard in pharmacovigilance
DP: design principle
DPA: disproportionality analysis
DSR: design science research
EMA: European Medicines Agency
FAERS: Food and Drug Administration Adverse Events Reporting System
FDA: Food and Drug Administration
HCI: human-computer interaction
II: information infrastructure
OHDSI: Observational Health Data Sciences and Informatics
ROI: return on investment
UMC: Uppsala Monitoring Centre
WHO: World Health Organization
