# Supplementary material for: Design Principles for Interactive Dashboards in Drug Safety Surveillance: Design Science Research
Source: JMIR Med Inform. 2026 Feb 27;14:e75936. doi: 10.2196/75936 (PMC13068636; doi:10.2196/75936)
Supplement: Multimedia Appendix 1 [file medinform-v14-e75936-s001.docx]

# Demographics of the Target Groups

**Table S1.** Demographics of the target groups.

| **ID** | **Type of participation** | **Attended sessions** | **Age** | **Gender** | **Country of residence** | **Professional experience** |
| --- | --- | --- | --- | --- | --- | --- |
| E1 | Expert | Co-design workshop, Heuristic Evaluation | 31 | Male | Germany | Data scientist, physicist, researcher in drug screening and development projects |
| E2 | Expert | Co-design workshop, Pilot evaluation, Heuristic Evaluation | 36 | Female | Germany | PhD in biochemistry, expertise in bioinformatics, data science, researcher in drug screening and development projects |
| E3 | Expert | Co-design workshop, Pilot evaluation, Heuristic Evaluation | 31 | Female | Germany | Human biologist, researcher in drug screening and development projects |
| E4 | Expert | Co-design workshop, Pilot evaluation | 34 | Male | Finland | PhD in biomedical engineering, data scientist, researcher in drug screening and development projects |
| C1 | Non-expert | Usability testing (think aloud session) | 28 | Female | Germany | Water and soil engineer |
| C2 | Non-expert | Usability testing (think aloud session) | 44 | Female | France | Lawyer, expertise in logistics deployment of medical humanitarian emergency response |
| C3 | Non-expert | Usability testing (think aloud session) | 28 | Male | Germany | Civil engineer |
| C4 | Non-expert | Usability testing (think aloud session) | 27 | Female | Sweden | PhD in biomedical engineering |
| C5 | Non-expert | Usability testing (think aloud session) | 42 | Male | Portugal | Data scientist |
| C6 | Non-expert | Usability testing (think aloud session) | 29 | Female | Portugal | Veterinary Student |
