# Supplementary material for: Design Principles for Interactive Dashboards in Drug Safety Surveillance: Design Science Research
Source: JMIR Med Inform. 2026 Feb 27;14:e75936. doi: 10.2196/75936 (PMC13068636; doi:10.2196/75936)
Supplement: Multimedia Appendix 2 [file medinform-v14-e75936-s002.docx]

# Design Science Research Approach Details

***Design Science Research Approach Phases***

The modified Design Science Research model phases used in this study are as follows:

(1) *Problem Definition* and *Objectives of the Solution*. We identified the problem space by reviewing recent literature on visual platforms for drug safety surveillance. The solution space was outlined in a response to the deficits of available platforms for pharmacovigilance and identified research gaps.

(2) *Diagnosis phase*. We identified a set of theory-driven, initial design principles. They were used as direct design recommendations in the prototype development. We performed the requirements gathering with a group of experts. As a support for this session, we used a v1-low-fidelity PowerPoint prototype.

(3) *Data Preparation phase*. We selected a suitable drug safety data source and chose five drugs for the prototype. We wrote python scripts to obtain and further process necessary data by querying the FDA database API. The scripts were also used for web scraping of DPA results.

(4) We performed three iterations of prototype development and its successive evaluation in a feedback loop. *Design and Development phase* is where we instantiated the v2-high-fidelity prototype using Tableau data visualisation software [1]. The choice of using Tableau as a proprietary software against existing open source choices stems out of the following criteria: (a) it offers superior and intuitive data visualization capability, is easy to use and has intuitive user interface navigation; (b) it helped accelerate co‑design with end‑users (addressing DP1a) and test core interaction affordances (selection‑keeping, overview–detail, attention cues) without building a custom front‑end.

Publishing via Tableau Public enabled open access for participants and readers, while all data and transformation code remain open‑source. For production or replication, the pipeline and design principles are portable to fully open‑source stacks (e.g., Apache Superset/Metabase for BI, or a custom React/D3 front‑end). In Table B1, we provide details of existing open-source alternatives.

(5) Each subsequent version of the prototype (v3-v4) was then evaluated in the *Demonstration and Evaluation phase*, where we performed a set of experimental sessions with users. Finally, in each iteration, we were revising the initial design principles to arrive at final, empirically-grounded DPs.

We discuss data collection in each phase in the following sections. Since design and development phases are indirectly concerned with data collection, we introduce them in sections related to iterations of data collection: diagnosis, data preparation, demonstration and evaluation phases.

## Diagnosis Phase – DPs and Requirements Gathering

Four experts participated in the session. The researcher was operating the computer with v1-low-fidelity PowerPoint prototype slides, where all suggestions were incorporated immediately in the form of comments. Individual elements were moved, reshaped, removed or added upon suggestion. Finally, two questions (*what is the best way to visually represent uncertainty of machine learning model prediction? Which dashboard features and elements, in your expert opinion, could facilitate the dashboard use by non-professional users?*) were put forward during the session to prompt informal discussion.

The session lasted two hours. We used the Otter.ai to record it [2].The software immediately transcribes recorded material and creates a separate audio for each participant. Transcripts were then shared in the common workspace (which participants also had an access to), cleaned, annotated, corrected and saved as final documents. Upon detailed analysis, data collected in the requirements gathering session was used for the development of the v2-high-fidelity prototype in Tableau.

We have chosen the FAERS database as a drug safety source as it is the only publicly available repository of pharmacovigilance data. Quarterly data files can be directly downloaded from the FDA website and used for the reconstruction of the database. FDA also offers data retrieval through API querying. Although the database is maintained by the U.S., it holds reports from across the globe (yet, the majority of them registered in the U.S. and Canada) [3].

## Getting the Data

We have selected five drugs for the prototype development. We motivate our selection in several ways: first, we looked for OTC drug classes generating the highest revenue between 2020 and 2022. This pointed to analgesics, and cold and cough remedies [4]. Second, we have written python code to query the FDA database to get the list of a hundred most commonly reported drugs (at the time of querying, the last database update was on 02.05.2022). Third, we considered requests from our participants on which drugs to include to make the dashboard insightful for them. Following these criteria, we have constrained the final drug list to: ibuprofen, acetaminophen (paracetamol), prednisone, quetiapine and morphine.

We adopted a modular pipeline that decouples data processing from visualization. Python ETL scripts ingest FAERS reports, normalize fields, add disproportionality metrics, thus outputting source‑agnostic exports. The dashboard reads only these denormalized tables. Scaling from a subset of drugs to all FAERS drugs, or extending the time window, requires recomputation of exports rather than changes to the visualization layer.

We retain FAERS‑native codings: adverse reactions are coded in MedDRA–a standard dictionary that can be translated to other vocabularies via persistent identifiers. Drug products are represented via the FAERS Product Dictionary derived from Structured Product Labeling (SPL) and the FDA Substance Registration System (UNII). UNIIs can be translated to RxNorm and ATC can be derived at the base‑ingredient level.

**Table S1:** Open-source alternatives.

| **Platform** | **Primary Focus/Strength** | **Notes** |
| --- | --- | --- |
| Apache Superset [5] | Data Exploration & Visualization | Offers a wide range of visualization options and an intuitive no-code viz builder, along with an advanced SQL IDE. Designed to be scalable. |
| Metabase [6] | Business Intelligence for Everyone | Known for its simplicity and user-friendly interface, making it accessible for both technical and non-technical users to ask questions and build dashboards (often without SQL). |
| Grafana [7] | Time-Series Data & Monitoring | Excels at visualizing time-series data, operational metrics, and monitoring, with robust alerting features and a large ecosystem of plugins and data source connectors. |
| Redash [8] | Collaborative SQL-Based Dashboards | Favored by SQL-savvy users. It's great for connecting to various data sources, writing queries, and creating and sharing dashboards collaboratively. |
| D3.js [9] | Highly Custom Data Visualization | A low-level JavaScript library. It's not a ready-to-use BI platform but offers unparalleled flexibility for developers to create unique, interactive web-based visualizations from scratch. |

**References**

1. Beard L, Aghassibake N. Tableau (version 2020.3). J Med Libr Assoc. 2021;109(1). [doi: 10.5195/jmla.2021.1135]

2. Liang S, Fu Y. Voice meeting notes & real-time transcription. Otter.ai. 2016. URL: <Otter.ai> [Accessed 2026-01-22]

3. EudraVigilance. European Medicines Agency. Jun 2022. URL: <https://www.ema.europa.eu/en/human-regulatory/research-development/pharmacovigilance/eudravigilance> [Accessed 2022-08-08]

4. Statista OTC pharmaceuticals - worldwide. Statista. 2022. URL: <https://www.statista.com/outlook/hmo/otc-pharmaceuticals/worldwide?srsltid=AfmBOoqOJV3dvb5VZaEczqUObuIS58T7fc9X1lQ43jIXDhkHj97tbJcK> [Accessed 2026-01-22]

5. Apache superset. Apache Software Foundation. URL: <https://superset.apache.org> [Accessed 2025-11-15]

6. Open source analytics that answers back. Metabase. URL: <https://www.metabase.com> [Accessed 2025-11-15]

7. Graphana labs. Graphana Labs. URL: <https://grafana.com/> [Accessed 2025-11-15]

8. Redash helps you make sense of your data. Redash. URL: <https://redash.io/> [Accessed 2025-11-15]

9. The JavaScript library for bespoke data visualization. D3. URL: <https://d3js.org/> [Accessed 2025-11-15]
