# Supplementary material for: Design Principles for Interactive Dashboards in Drug Safety Surveillance: Design Science Research
Source: JMIR Med Inform. 2026 Feb 27;14:e75936. doi: 10.2196/75936 (PMC13068636; doi:10.2196/75936)
Supplement: Multimedia Appendix 3 [file medinform-v14-e75936-s003.docx]

# Prototype in Tableau

Figures S1-S4 represent the version v2-v3 of the prototype, whereas figures S5-S8 represent the version v4 of the prototype.


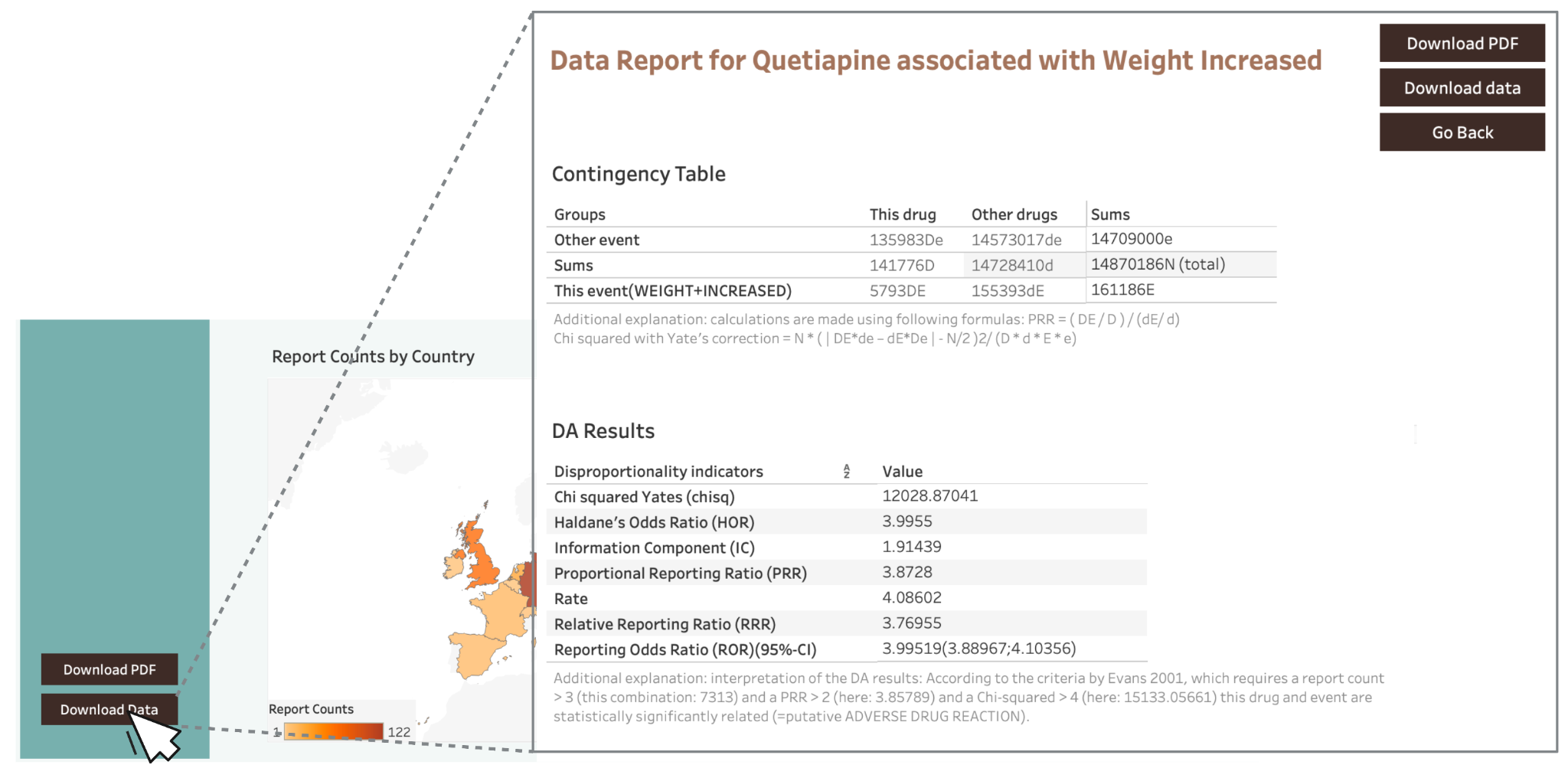
**Figure S1.** The first Tableau (v2) prototype: clicking on the *Download Data* button (bottom left) in the main dashboard opens the safety signal report page with DPA metrics (right). Multiple download buttons (upper right and bottom left).


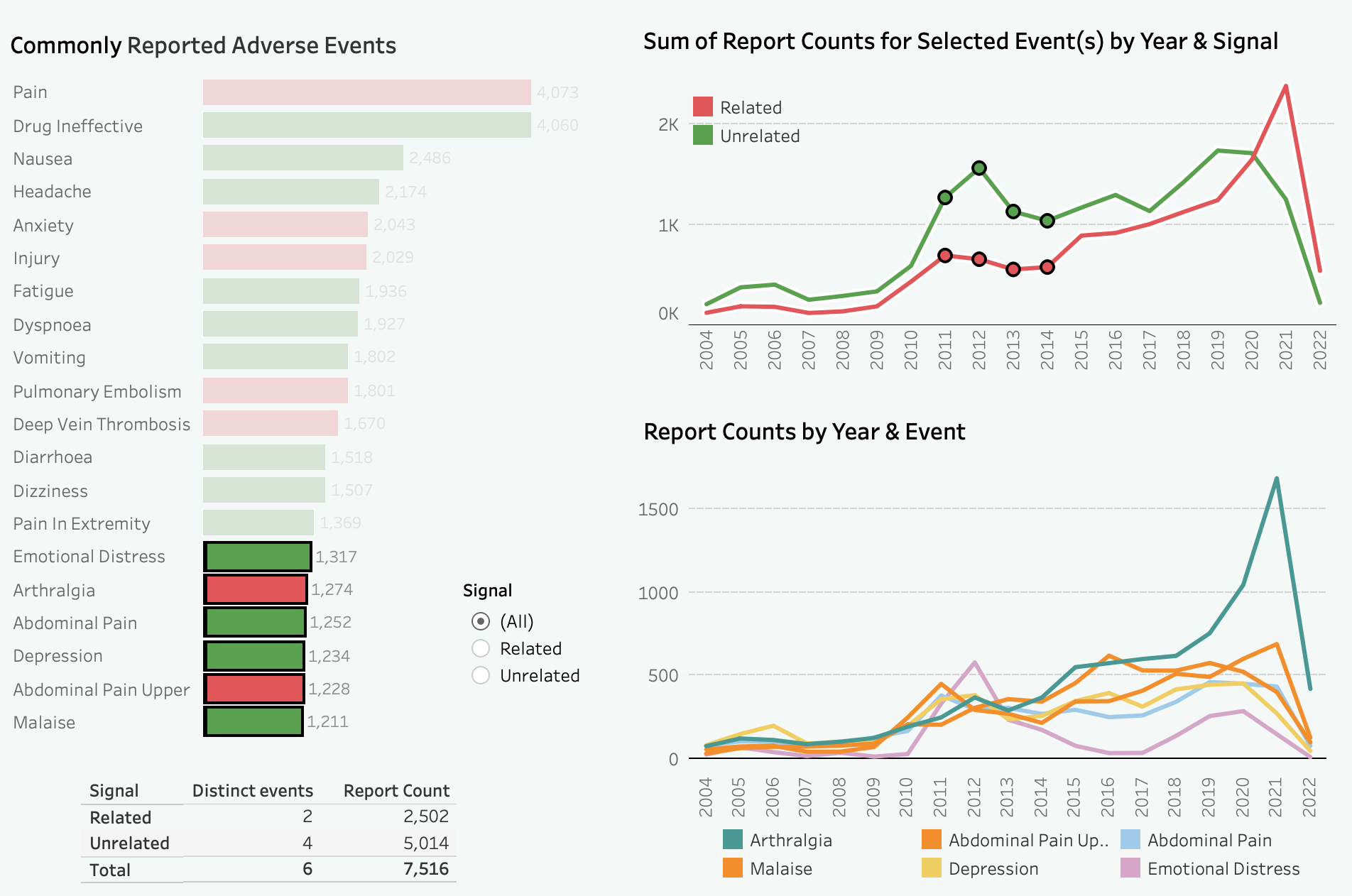


**Figure S2.** The first Tableau (v2) prototype: interactivity of the dashboard – selection of bars on histogram (left) influences ADRs displayed in the second line graph (bottom right). Selecting year ranges on the first line graph (upper right, circled year values) influences the histogram, changing the count and distribution of ADRs.


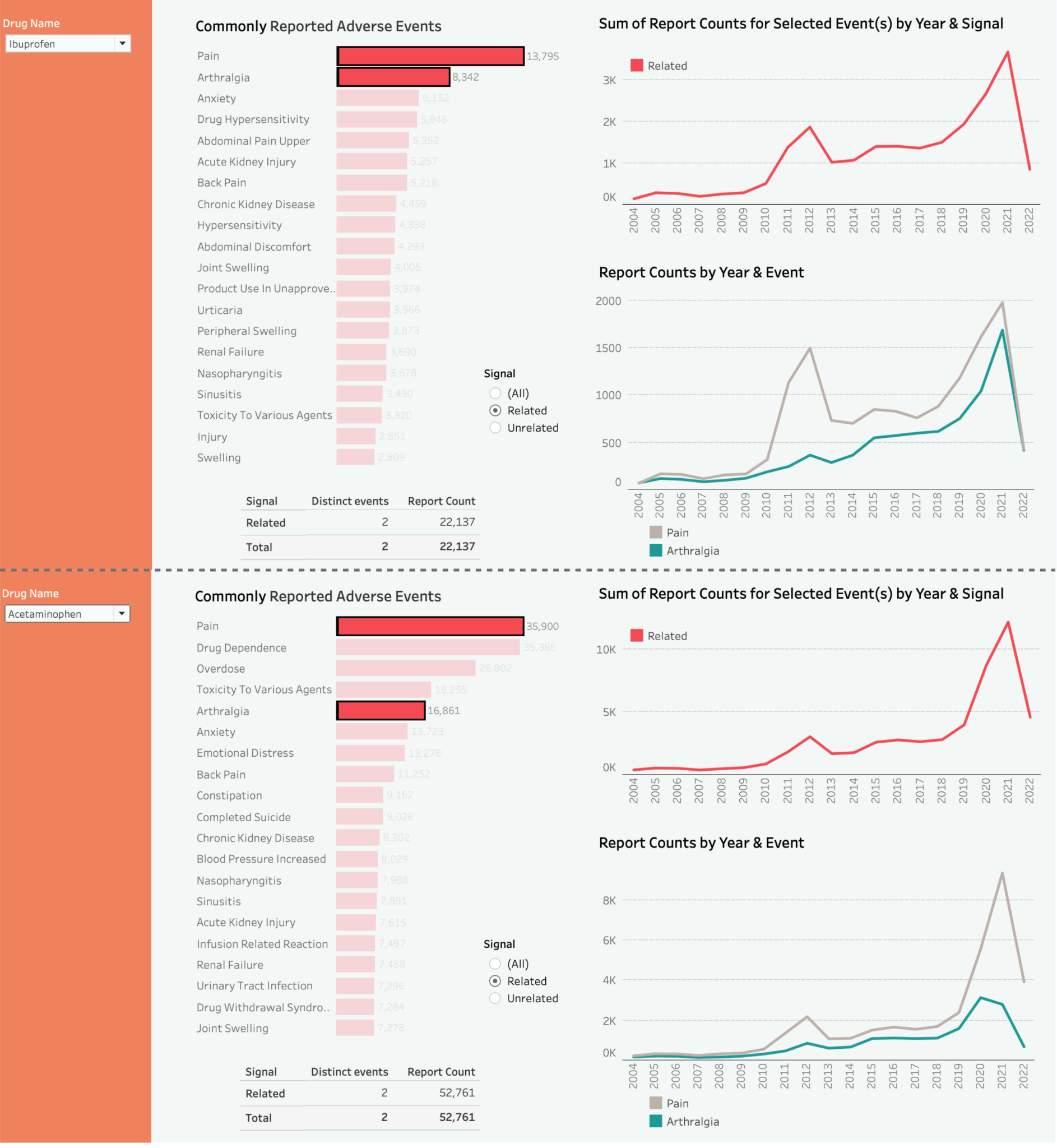
**Figure S3.** *Selection-keeping* feature. Prototype v3.

In Figure S3, safety profiles of two common analgesics – Ibuprofen (top) and Acetaminophen (Paracetamol) (bottom) are compared. *Pain* and *Arthralgia* have been evaluated as statistically significantly related to both drugs. The first line graph (upper-right corners) displays the sum of report counts for *Pain* and *Arthralgia* for each drug. The second line graph (lower-right corners) displays the distribution of report counts for *Pain* and *Arthralgia* separately (for each drug). This allows detailed exploration of data, e.g., it is clear that the increase in report counts for Acetaminophen in 2021 was due to the high number of reports for *Pain*, rather than *Arthralgia*, which is not the case for Ibuprofen. Caution in interpretation of the results is necessary: over 30 thousand reports of *Pain* with Acetaminophen versus over 13 thousand reports for *Pain* with Ibuprofen does not mean that Acetaminophen is more likely to cause *Pain*. Such results can be related to differences in the total number of reports for each drug (over 400 thousand for Ibuprofen and over 900 thousand for Acetaminophen, not shown in the figure). Thus, the examination of detailed DPA metrics (included in downloadable files) is necessary to formulate hypotheses on safety profiles of each drug.


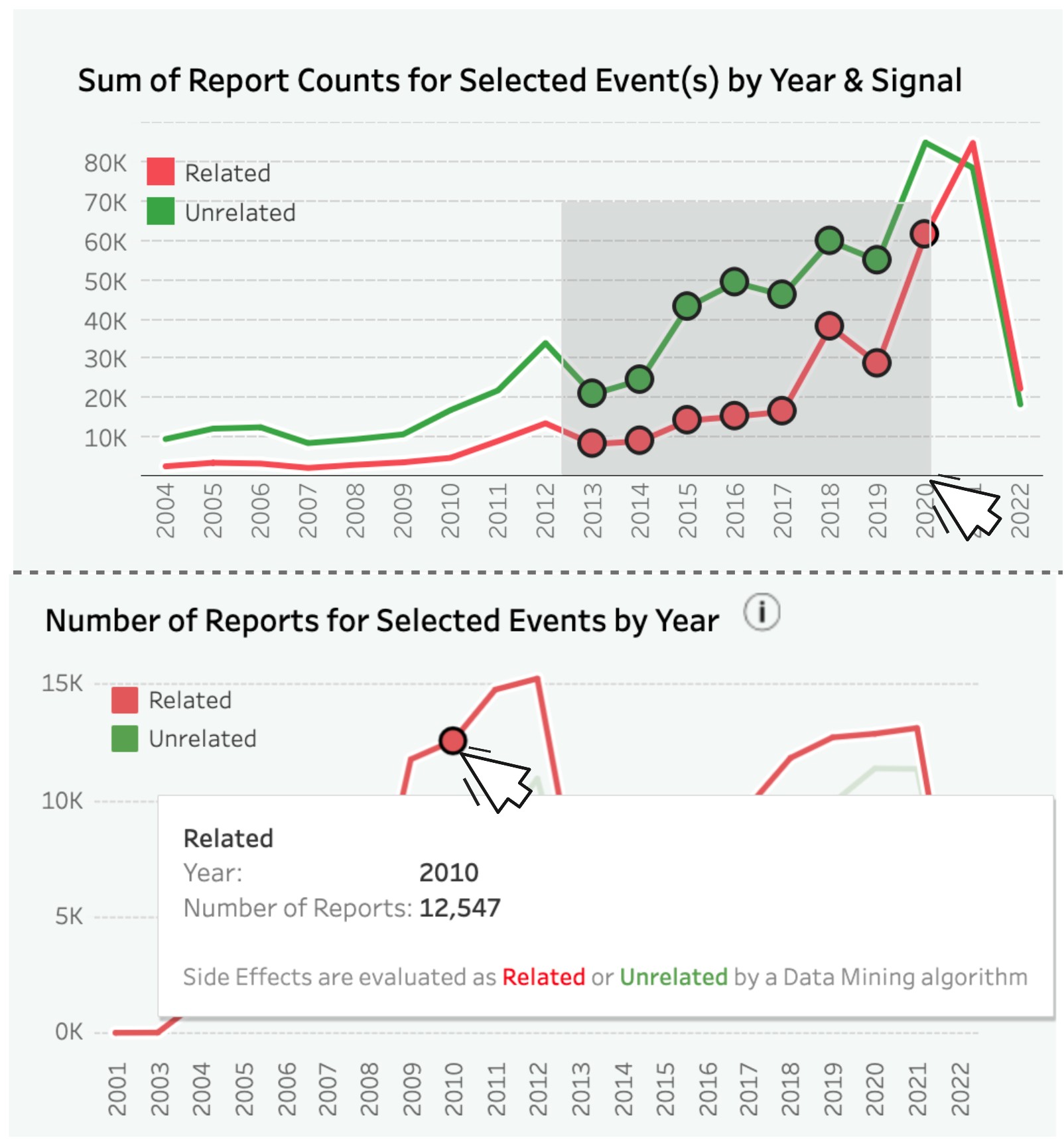


**Figure S4.** The first line graph and possible ways of selecting a year range (by dragging the section with the mouse, top) or clicking year values directly on the line in-chart (bottom). Selection by clicking on x-axis labels was also possible (not shown). Prototype v3.

#


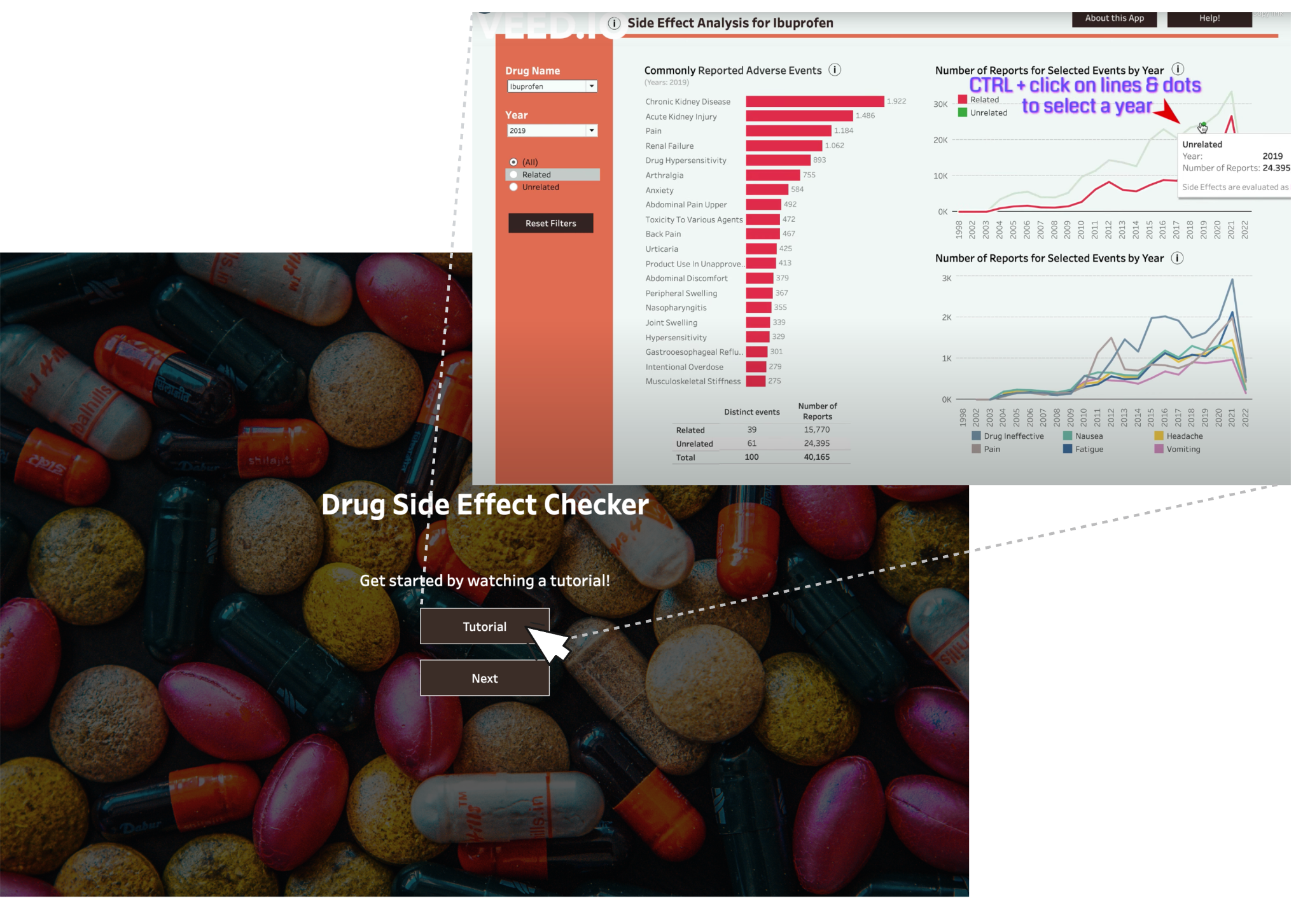
**Figure S5.** The landing page. Users can choose to watch the *getting started* video tutorial or move directly to the dashboard (the *Next* button). Exemplary video tutorial frame (upper right). Prototype v4.


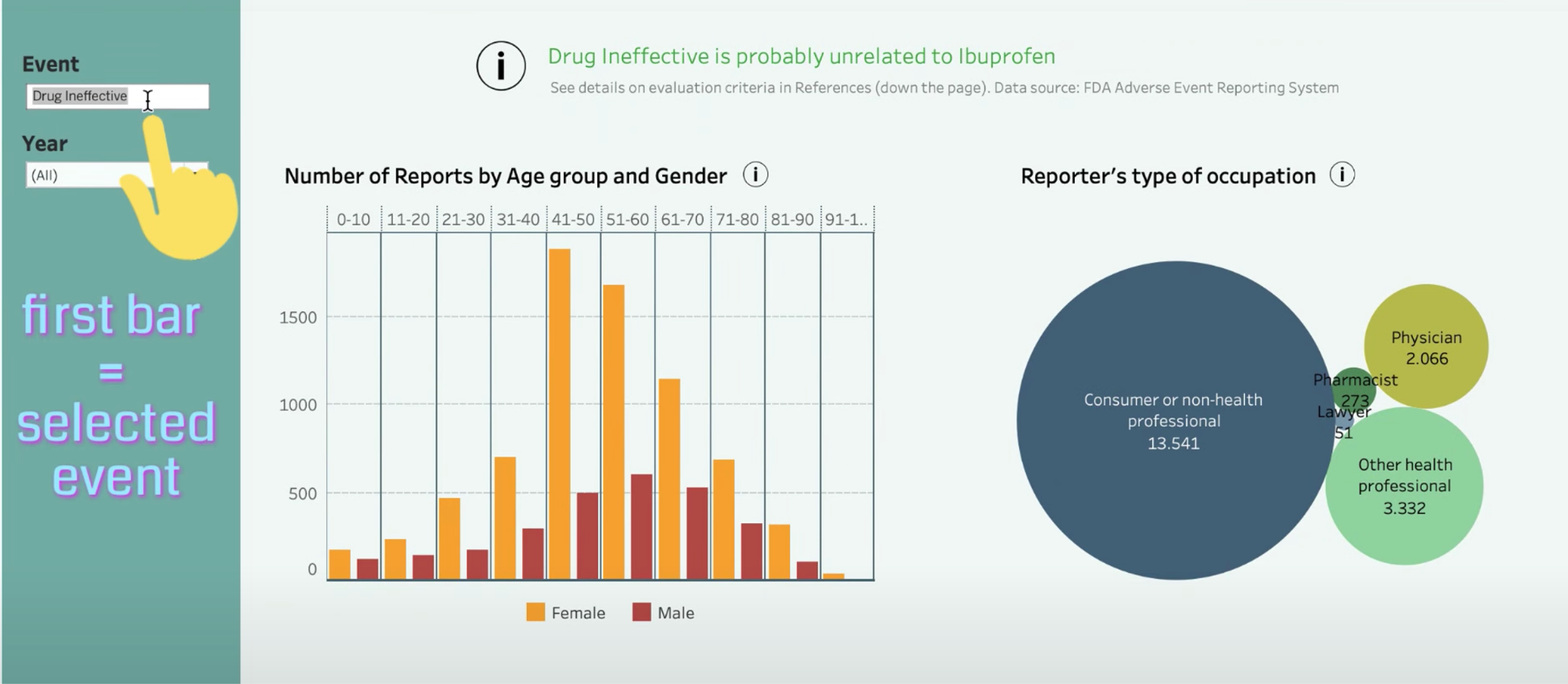
**Figure S6.** The functionality of selecting ADRs by clicking on histogram bars was explained in the *getting started* video tutorial. Prototype v4.


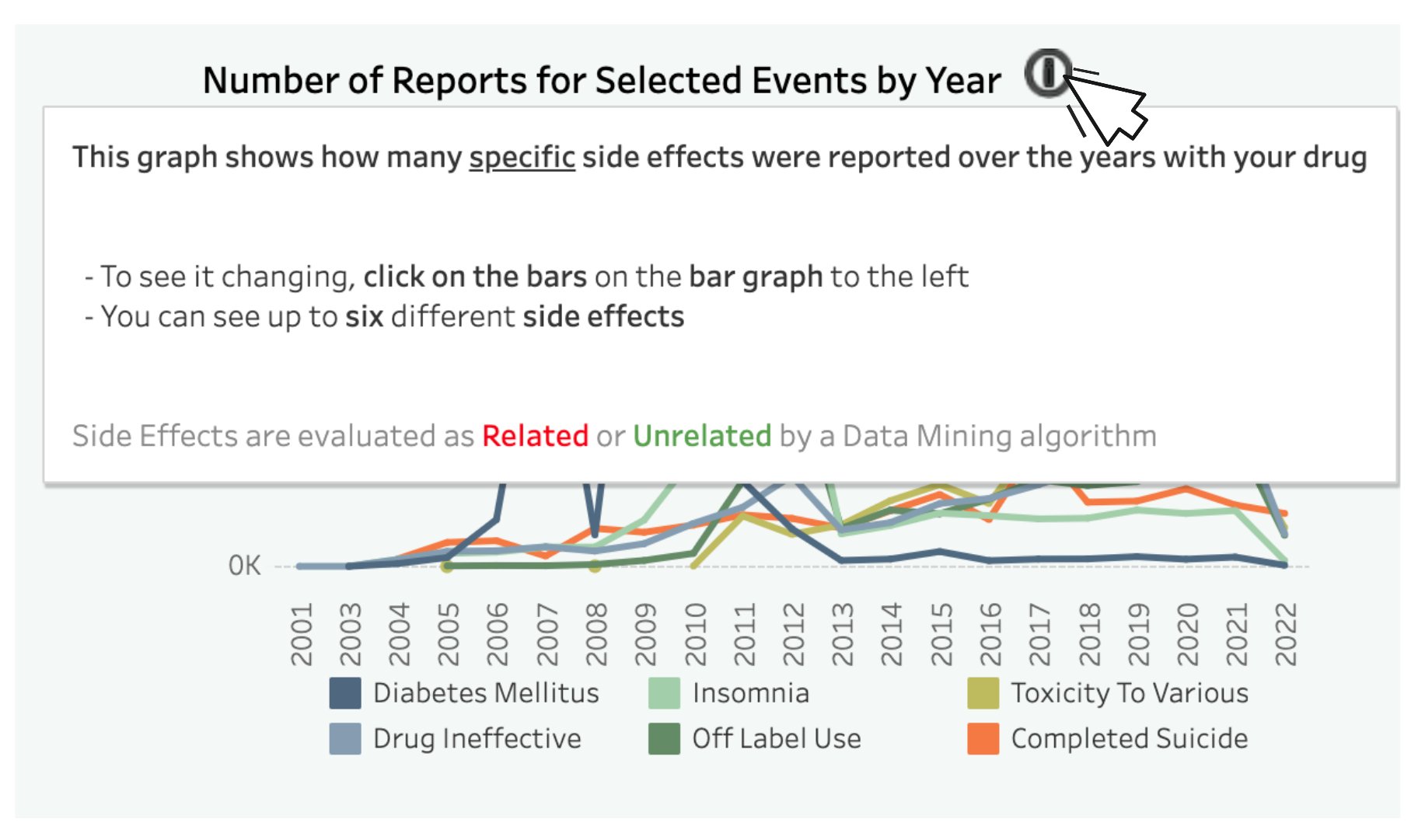
**Figure S7.** The utility of the second line graph was signalised through the short label with essential information. Hovering with the mouse over the info icon makes the label appear. Prototype v4.


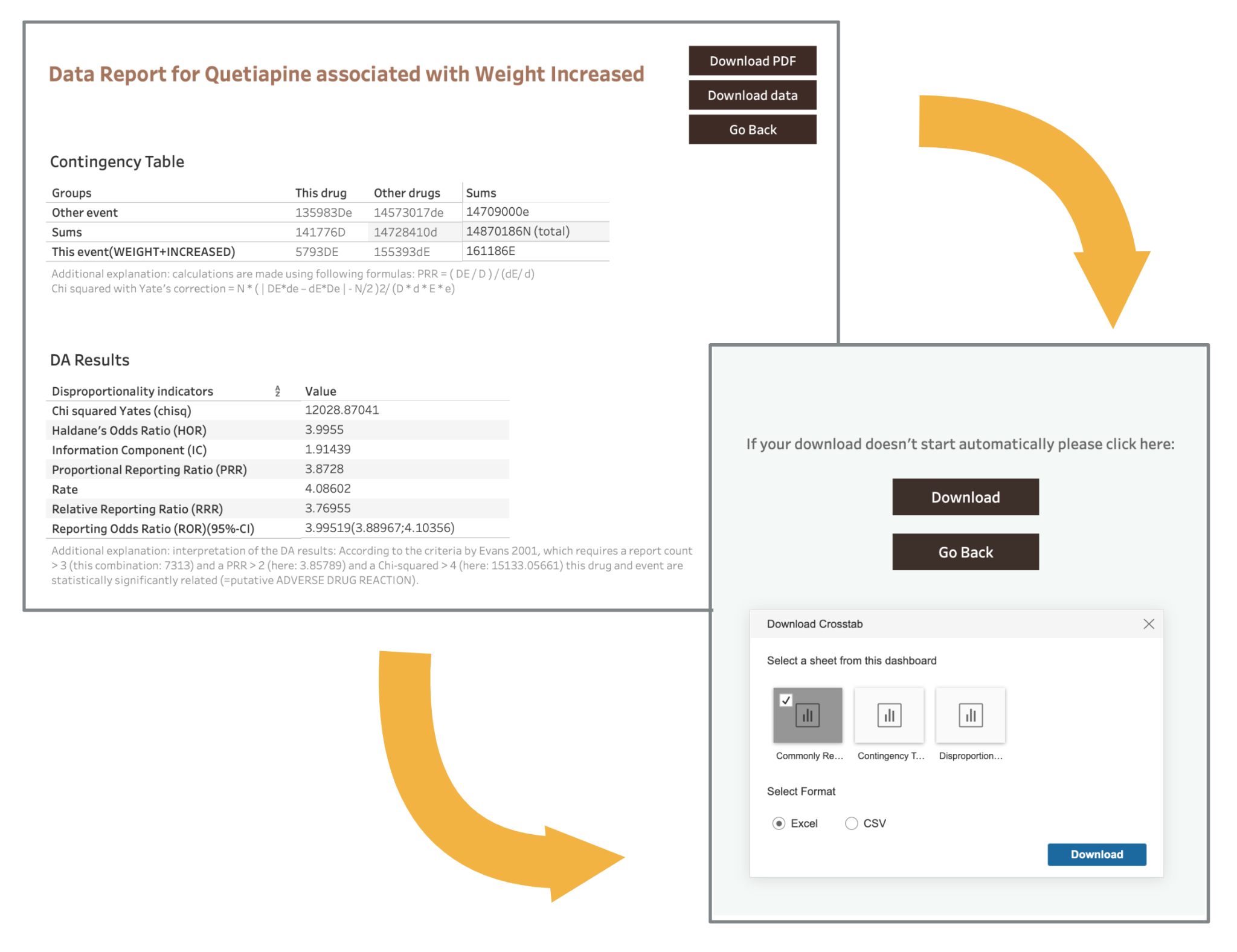


**Figure S8.** A comparison of the previous download page with multiple buttons and safety signal report in the background (upper left) and current, modified download page with a single download button (bottom right). Prototype v4.
