# Supplementary material for: Design Principles for Interactive Dashboards in Drug Safety Surveillance: Design Science Research
Source: JMIR Med Inform. 2026 Feb 27;14:e75936. doi: 10.2196/75936 (PMC13068636; doi:10.2196/75936)
Supplement: Multimedia Appendix 4 [file medinform-v14-e75936-s004.docx]

# Usability Testing and an Overview of Solutions Implemented

**Table S1.** An overview of the findings in usability testing sessions. Field Solution ID provides the link to Table D2 below – an overview of implemented solutions. Through Solution ID, it is possible to track all solutions that have been assigned to each usability issue. In most of the cases, we responded to usability issues by implementing more than one solution. Design principles provided in this table relate to the corresponding feature or element of the dashboard, and not to the usability issue or assigned solution.

| **Feature or element** | **Usability Issue** | **Related DPs** | **Solution ID** |
| --- | --- | --- | --- |
| *Getting started* tutorial | The text is long, and the screenshots are too small, making them difficult to read or understand. Some concepts are unclear. The tutorial is lengthy, and users tend to quickly click through it using the "next" button. The absence of a skip button adds to the frustration, as users cannot bypass the tutorial. There is no option to revisit the tutorial once it has been completed. | DP 1a (*designing for direct use*) | 27, 3, 52 |
| Histogram filter | The filter’s name is confusing (s*ignal*) for the users who did not understand its connection to events *related* or *unrelated* to the drug. They struggled to locate the filter on the dashboard and often overlooked the information explaining how side effects *relate* or *do not relate* to the drugs in question. | DP 2b (*overview and details*), DP 2c (*attention stimulation*), DP 2a (*customised views*), DP 1b (*causality between variables*) | 52, 3, 4, 36, 6, 7, 43, 42 |
| First line graph | The methods for selecting individual years or year ranges were unclear. Many users were unaware of the option to drag with the mouse and thought it was necessary to click each year individually on the x-axis. Additionally, some users did not realise they could use the individual lines (red for *related* events, *green* for unrelated events) to modify their selection. Some users took a long time to understand how to remove the selection. Some have selected unwanted ranges and could not find the way back. | DP1c (*state-tracking*), DP 2a (*customised views*) | 52, 3, 9 |
| Second line graph | Most users either disregarded the second line graph or were uncertain about its purpose and how to use it. | DP1c (*state-tracking*), DP 2a (*customised views*) | 52, 3, 40 |
| Histogram | Users were unaware that they could select individual side effects by clicking on the histogram bars; they were using y-axis labels to do so. Users did not understand how to select by *dragging* the sections with the mouse. | DP 2b (*overview and details*), DP 2c (*attention stimulation*), DP 2a (*customised views*), DP 1b (*causality between variables*) | 52, 3 |
| Year filters and year selection | The lack of the year selection filter in *overview* sections of the dashboard was confusing. Users found it challenging to determine which years were selected, and there was no option to remove the selection. | DP 2b (*overview and details*), DP 2a (*customised views*) | 52, 3, 1, 2, 9 |
| Age-gender distribution graph | Users expected to see the sum of report counts for males and females on the graph. Users were confused with the limited interactivity of this graph (and related sections). | DP 2b (*overview and details*), DP 3b (*decision-making support*), DP 2c (*attention stimulation*) | 16, 52 |
| Event search field | Users found the position and the visibility of the event search field unideal. Some users were confused by the fact that their selection of bars on the histogram affected the side effect selection in the event search field. | DP 2b (*overview and details*) | 31, 52, 3 |
| Download buttons and DPA metrics files | Users were confused with the presence of more than one download button. Upon clicking the button, the dialog box was not in the eye reach and the users were expecting the download to start automatically. The presence of multiple DPA files further added to the users' confusion. | DP 3b (*decision-making support*), DP 2b (*overview and details*) | 23, 25, 33, 44, 34 |
| Safety signal reports | Detailed safety signal reports (displayed in the background upon clicking the *download* button) are intended to target mainly professional users and provide unnecessary information overload for non-professional users. | DP 3b (*decision-making support*), DP 2b (*overview and details*) | 30 |
| Nomenclature | Users were confused with graphs or variable names on several occasions. This had a negative impact on their task performance. | DP 1a (*designing for direct use*) | 55 |

**Table S2.** An overview of implemented solutions (after usability testing). Solution ID links this table to Table D1 – an overview of the findings in usability testing. ROI (Return on Investment) is a cost-benefit ratio of each solution.

| **Solution** | **Solution ID** | **Return on Investment (ROI)** |
| --- | --- | --- |
| Modify the *getting started* tutorial, so that it includes less text and is animated. Essentially, change the tutorial type to an app walkthrough video. | 27 | 10.2 |
| Explain all dashboard elements and functionalities (what is their purpose, what is possible with them and how the users benefit from their usage). Add information on how to interact with dashboard elements. | 52 | 26.3 |
| Create a *Help* button to go back to the tutorial. | 3 | 107.7 |
| Create an informative box *About this App* | 4 | 24.9 |
| Add short and understandable explanation behind the meaning of *related* and *unrelated* events | 36 | 5.7 |
| Add an information about current year selection | 2 | 16.5 |
| Add year filter (affecting the histogram and both line graphs) to *up the page* section (where *overview* is shown) and place it in visible area | 1 | 24.4 |
| Change the name of the histogram filter (selection of related, unrelated or all events) | 6 | 12 |
| Relocate the histogram filter | 7 | 6.7 |
| Create a *remove selection* button | 9 | 5.4 |
| Add information about the sum of report counts for male and female | 16 | 5 |
| Change the naming of download buttons | 23 | 25 |
| Remove the *download pdf* button | 25 | 12.5 |
| Remove the option of pdf download (downloads the screenshot of entire dashboard) and keep only the option of detailed DPA metrics files download | 33 | 25 |
| Remove less important files from download option to increase the readability of download section | 44 | 14.2 |
| Remove the safety signal reports displayed in the background upon *download* buttons clicking. Keep this information in downloadable files instead (as it targets professional users) | 30 | 50 |
| Increase font size for search field titles (*Event* and *Drug name*) to make them more visible | 31 | 16.2 |
| Improve the visibility and utility of the second line graph | 40 | 5 |
| Emphasise the information on the data source (in warning messages and in dashboard header) | 42 | 12 |
| Change the subtext in Warning Text box to more informative and simpler, and implement *references* button (e.g., *See details on the evaluation criteria in references*) | 43 | 16.9 |
| Modify graph and variable names wherever necessary | 55 | 9.5 |
| Relocate download dialog box, so that it is in user’s eye reach | 34 | 12.5 |
